# Why Do PETases Struggle with Crystalline PET? Catalytic Ensemble Sampling Reveals Molecular Bottlenecks

**DOI:** 10.1021/acs.jpclett.6c00308

**Published:** 2026-04-16

**Authors:** Ania Di Pede-Mattatelli, Miguel A. Maria-Solano, Oleksandr Haisha, Francesco Colizzi

**Affiliations:** † Molecular Ocean Lab, Institute for Advanced Chemistry of Catalonia, IQAC−CSIC, Carrer de Jordi Girona 18-26, 08034 Barcelona, Spain; ‡ Molecular Ocean Lab, Institute of Marine Sciences, ICM−CSIC, Passeig Marítim de la Barceloneta 37-49, 08003 Barcelona, Spain; § Graduate Programme in Biotechnology, Universitat de Barcelona, Gran Via de les Corts Catalanes 585, 08007 Barcelona, Spain

## Abstract

Polyethylene terephthalate (PET) is a widely used thermoplastic whose high crystallinity poses a major barrier to upscaling enzymatic recycling. Although PETases with high activity and stability have been reported, no enzyme has been shown to directly depolymerize crystalline PET (cPET), and the molecular determinants limiting their efficacy remain challenging to characterize. Here, we integrate experimental conformational ratios of crystalline and amorphous PET chains with enhanced-sampling molecular dynamics simulations to map the free-energy landscape of a prototypical PETase bound to PET oligomers, revealing how structural equilibria translate to catalytic function. We find that productive enzyme–substrate catalytic configurations can be reached for both crystalline and amorphous PET chains. However, the formation of catalytic ensembles on cPET is strongly hindered by the enzyme shape and dynamics, with additional energetic costs required to separate crystalline chains to fit the active site, consistent with experimental data. The model highlights limitations of the active-site architectures typically found in α/β-hydrolase scaffolds used for PET depolymerization and indicates directions for their redesign to enable cPET degradation. Our approach showcases a general strategy to leverage the quantitative characterization of catalytic ensembles for interrogating substrate–enzyme interactions and informing molecular design.

## Graphical abstract

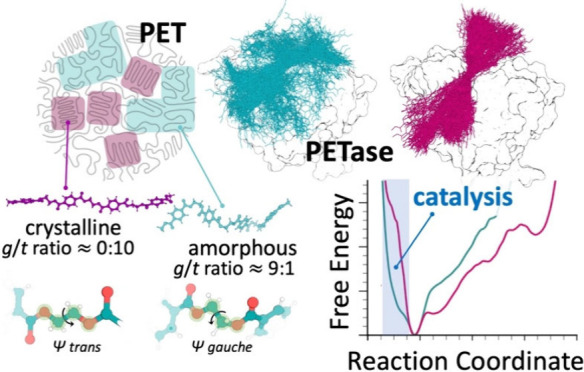

Plastics are essential to modern life, yet improper disposal has kept global recycling rates below 30%, with most plastic waste incinerated, landfilled, or leaking into natural ecosystems at a staggering rate.^1,2^ Enzymatic depolymerization of polyethylene terephthalate (PET) offers a prototypical strategy to address these recycling challenges, as its C–O ester bonds can be cleaved by polyester hydrolases (PET hydrolases or PETases), enabling conversion of plastic waste and monomer recovery.^3,4^ Although PETases with high hydrolytic activity and stability have been widely reported in the literature,^3,5^ key challenges remain before their full industrial exploitation can be realized.^4,6^ A major obstacle to cost-efficient enzymatic PET recycling is the high crystallinity typical of beverage bottles and textile fibers, which renders the polymer largely resistant to enzymatic attack. Consequently, costly pretreatments such as thermal or chemical amorphization are often required.^3^ Indeed, in semicrystalline thermoplastics like PET, enzymatic degradation predominantly occurs in the mobile amorphous regions, as the tightly packed chains in crystalline domains are generally inaccessible to the enzyme (Figure 1).^7^ Despite extensive research,^8,9^ the molecular basis modulating the access of the PETase active site to the reactive centers of crystalline PET and the factors limiting the enzymatic efficiency in such regions are still not fully resolved.

**Figure 1:**
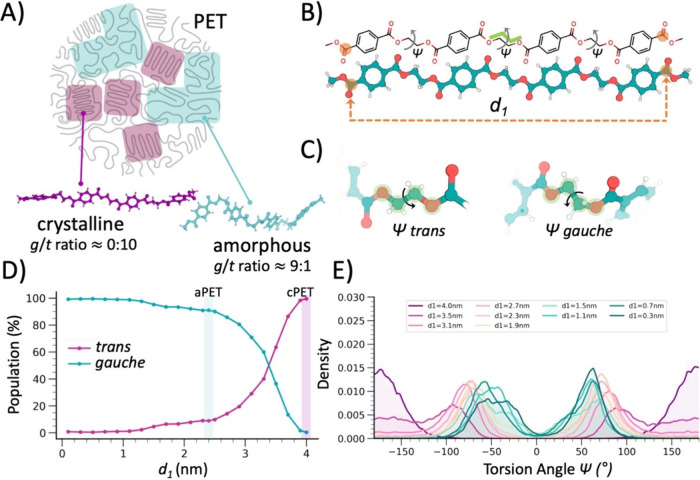
Modeling amorphous and crystalline polyethylene terephthalate (PET) oligomers by matching experimental *gauche*/*trans* (*g*/*t*) ratios. (A) Schematic model of semicrystalline PET. Polyester hydrolases degrade, to different extents, the accessible amorphous fractions (in cyan), while the crystalline fractions (in violet) are recalcitrant to enzymatic hydrolysis. At the bottom: molecular snapshots of amorphous and crystalline PET chains, with the corresponding *g*/*t* ratios matching the experimental values. (B) Chemical representation of a PET tetramer with the torsion angle Ψ highlighted. The terminal atoms used to define the minimum distance *d*_1_ are highlighted in orange. (C) Close up on the EG torsion angles (Ψ; OC–CO, showing *trans* and *gauche* conformations. (D) Population of *trans* and *gauche* Ψ conformations sampled during the simulations, plotted as a function of the distance (*d*_1_). (E) Conformational distribution of the EG torsion angles Ψ for different values of the minimum distance (*d*_1_).

At the molecular level, amorphous PET exists as a mixture of *trans* (*t*, ∼180°) and *gauche* (*g*, ∼70°) conformers of the torsion angle (Ψ) OC–CO in the ethylene glycol (EG) moiety, with the *gauche* form typically predominating (91:9 *g*/*t* ratio at 30 °C).^10^ In contrast, crystalline PET adopts a fully *trans* conformation (Figure 1A).^11,12^ Previous studies have shown that optimizing the conformational match between the polymer and the enzyme is key to improving biocatalytic degradation efficiency.^13,14^ Unlocking the ability of enzymes to directly depolymerize crystalline PET remains a key research objective,^4,15–17^ with the potential to deliver a major leap in the sustainability and economic viability of large-scale PET recycling.^3,4^ Achieving this goal will likely require integrating enzyme design with a molecular-level understanding of enzyme interactions with both amorphous and crystalline PET. While experimental techniques are beginning to provide insights into enzyme–polymer interactions for amorphous PET,^18–21^ crystalline PET remains largely inaccessible to these methods. In this context, molecular dynamics (MD) simulations, capable of modeling any PET state under controlled microscopic conditions, represent a powerful and complementary approach. When coupled with enhanced-sampling and free-energy methods, MD offers unique insight into the mechanisms and energetics underlying the biomolecular processes involved.

Recent insight has been generated from MD simulations in synergy with experimental data, offering a general blueprint of the dynamics of PETases with amorphous PET melts, under a variety of conditions.^8,9,22–24^ More recently, this analysis has been extended to crystalline PET surfaces,^9^ and quantum mechanical calculations have begun to address the estimation of energy barriers involved in crystalline PET hydrolysis, starting from binding modes generated by rigid molecular docking.^25^ MD simulations were also used to examine single-chain decrystallization.^26^ Although highly informative, these studies have provided mostly a qualitative description of the link between PET binding and the formation of productive enzyme–substrate configurations for catalysis, leaving challenging questions about the mechanisms and free-energy landscapes underlying crystalline and amorphous PET depolymerization unresolved.

To address these challenges, here we devised an enhanced-sampling MD approach to reconstruct the free-energy landscape of idealized crystalline and amorphous PET oligomers bound to PETase. We leverage experimentally known *g*/*t* ratios of PET chains in crystalline and amorphous states to reduce the complexity of PET–enzyme interactions, thereby focusing specifically on the free-energy region surrounding productive catalytic configurations. This enables us to expand and manipulate PET–enzyme structural ensembles in ways consistent with, and beyond, experiments. We focus on *Ideonella sakaiensis* PETase (*Is*PETase)^27^ as a prototypical aPET depolymerizing enzyme, which—like all known PETases—is inactive against crystalline PET.^4,28^ We find that the free-energy cost of forming productive catalytic configurations for crystalline compared to amorphous PET is strongly penalized by the structure and dynamics of the hydrolase active site. Combined with the additional cost of separating tightly packed cPET chains, this drastically limits enzyme function on crystalline substrates. The results provide a quantitative framework to address ensemble-based active site redesign—potentially embedded in novel protein scaffolds—toward efficient cPET depolymerization. More broadly, this approach offers a conceptual strategy to probe enzyme–substrate catalytic ensembles in semicrystalline plastics.

## Modeled PET Conformations Match Experimental *g*/*t* Ratios

We modeled crystalline and amorphous PET oligomers by reproducing the conformational probability distribution of longer polymeric substrates observed experimentally by NMR (Figure 1).^10–12^ In particular, we modulated the different *g*/*t* ratios of the EG torsion angle (Ψ; OC–CO) by introducing a restraining potential that limits the region of phase space accessible during the MD simulation, thereby effectively modulating the minimum distance (*d*_1_) between the termini of a PET oligomer composed of four subunits (Figure 1B, 1C). Depending on the values of *d*_1_, a diverse range of *g*/*t* ratios was observed (Figure 1D, 1E). Specifically, at a *d*_1_ value of 2.3–2.4 nm, a *g*/*t* ratio of 9:1 was obtained, with a large peak at about ± 70° and a smaller one near 180°, consistent with the probability distribution of the torsion angle Ψ in commercially available amorphous PET, where a *gauche*-to-*trans* ratio of 91:9 is reported at 30 °C.^10^ In parallel, at a *d*_1_ of 4.0 nm, a 100% *trans* conformation of Ψ was observed in our simulations, corresponding to the fully *trans* content of crystalline PET as reported in two-dimensional NMR patterns of *trans* and *gauche* conformations.^11,12^ Based on these observations, we selected *d*_1_ values of 2.4 and 4.0 nm to model idealized chains of amorphous and crystalline PET, respectively.

## Crystallinity Hinders the Formation of Catalytically Competent Enzyme–PET Ensembles

To probe the capability of *Is*PETase to interact with these cPET and aPET substrates and quantify the energetics of the possible catalytic ensembles involved, we combined fully atomistic well-tempered Metadynamics (wt-MTD)^29,30^ with Hamiltonian Replica Exchange (HREX, in its REST2 variant)^31–34^ MD simulations. This approach enabled us to characterize the free-energy landscape of the system in proximity to the corresponding catalytic ensemble, while simultaneously enhancing the conformational heterogeneity of the PET oligomer (Figure 2; see Methods and the Supporting Information, SI).

**Figure 2:**
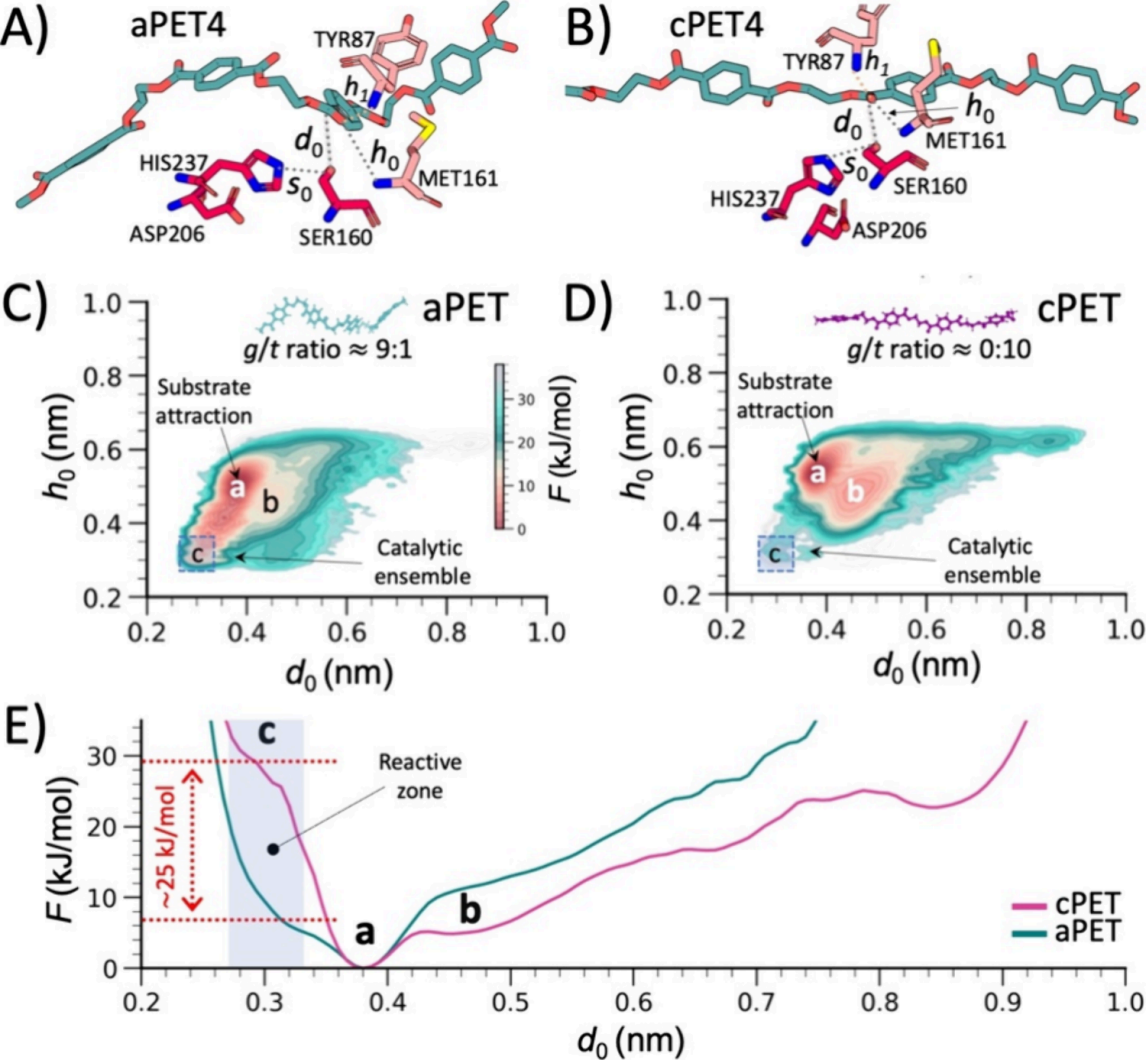
Free-energy landscape of *Is*PETase–PET systems in proximity of the catalytic ensemble. Molecular model of the enzyme–PET complex and the reaction coordinate or collective variables (CVs) used for HREX-wt-MTD analysis and reweighting with (A) amorphous and (B) crystalline PET chains. Free-energy surfaces of *Is*PETase bound to (C) amorphous and (D) crystalline PET, shown as functions of the *d*_*0*_ and *h*_0_ distances. (E) Free-energy profiles projected onto the *d*_0_ distance (between the Oγ of catalytic Ser160 and the PET carbonyl carbon) for amorphous (cyan) and crystalline (violet) PET chains. See Supplementary Figure S2 for additional projections and block-analysis error estimates.

The free-energy landscape was reconstructed as a function of several key distances characterizing the catalytic ensemble (highlighted in Figure 2A, 2B and Supplementary Figures S2 and S7). To guide the discussion that follows, it is important to distinguish between two types of free-energy differences: (i) small local variations between neighboring basins, and (ii) the larger free-energy penalty required for crystalline versus amorphous PET to adopt catalytically competent configurations, i.e., the energy needed to move from basin “a” to basin “c” in the profiles shown in Figures 2C–E.

Projecting onto the distances *d*_0_ and *h*_0_, corresponding respectively to the distance between the PET carbonyl carbon and Ser160 Oγ, and the distance between the PET carbonyl oxygen and the backbone nitrogen of Met161, we observed a broad basin of substrate attraction (basin “a” in Figures 2C–D) for both aPET and cPET, centered at *d*_0_ ≈ 0.39 nm and *h*_0_ ≈ 0.55 nm. At these positions, the substrate is not yet engaged with the oxyanion hole (backbone NH groups of Met161 and Tyr87, Figures 2A–B), highlighting a first step in the approach toward a catalytically competent configuration. In the aPET–PETase system, this basin “a” well connects to a higher free-energy region (basin “c” in Figure 2C, 2E), about 5 kJ/mol above, where *d*_0_ and *h*_0_ reach ∼0.3 nm, distances compatible with a catalytically competent ensemble. These configurations are consistent with those required for nucleophilic attack, as proposed by recent quantum mechanics/molecular mechanics (QM/MM) studies^35–37^ and multiscale QM/MM investigations^38^ on substrate–enzyme reactivity.

In contrast, when *Is*PETase interacts with cPET, the wide substrate attraction basin connects to an adjacent free-energy region that is also about 5 kJ/mol higher but where the catalytic distance *d*_0_ is shifted to ∼0.45 nm (label “b” in Figure 2D, with *h*_0_ ∼ 0.45 nm), thus deviating from configurations compatible with catalysis. Scanning the free-energy profile along *d*_0_, we find that configurations with short *d*_0_ distances—ideally compatible with catalysis—are accessible, but they lie approximately 25–30 kJ/mol above the main free-energy minimum (Figure 2E, violet plot). When comparing aPET and cPET along this principal reaction coordinate (*d*_0_), we observe a difference of approximately 25 kJ/mol in the system ability to populate catalytically competent configurations required for substrate hydrolysis (Figure 2E, red arrow). This energetic penalty in the reactant state of cPET relative to aPET reduces the population of catalytically competent complexes by ∼24 000-fold (calculated as *e* ^–Δ*F*/*k*_B_ *T*^, where Δ*F* is the free-energy difference, *k*_B_ the Boltzmann constant, and *T* the temperature). This drastic depletion effectively eliminates productive cPET-bound catalytic ensembles, in line with the near-complete loss of enzymatic activity. By comparison, literature shows that even a few orders-of-magnitude decreases in the population of catalytically competent substates can lead to substantial activity drops.^39,40^

Furthermore, we observed that the substrate conformational state modulates the stability of the hydrogen bond between Ser160 and the adjacent His237, which is essential for Ser160 activation for nucleophilic attack (distance s_0_; Figures 2A–B and Supplementary Figures 2F and 2O). In particular, in the presence of cPET, the Ser160–His237 hydrogen bond is approximately 6 kJ/mol less stable than in the aPET complex (Supplementary Figures 2F and 2O).

To better understand what limits PETase access to the reactive center of cPET, we projected the free-energy landscape onto collective variables that describe enzyme–substrate interactions in more detail (Figure 3). Trp185 is known to modulate substrate binding and positioning through the “wobbling” conformations observed in several *Is*PETase X-ray crystal structures.^41,42^ The χ_2_ angles adopted by Trp185 in these structures^41^ span a broad range of values (Figure 3A, dashed gray lines), and mutation studies indicate that this high flexibility is functional for binding and depolymerizing aPET.^41,42^ Motivated by this observation, we examined the conformational behavior of Trp185 in the presence of aPET and cPET chains. The two substrate types modulate the χ_2_ conformational landscape of Trp185 in markedly different ways. In aPET complexes, the free-energy minima leading to reactive *d*_0_ distances include the wobbling χ_2_ orientations seen in experimental structures^41^ (Figure 3A, dashed gray lines). In contrast, when bound to cPET, Trp185 samples not only the wobbling states—in this case incompatible with productive *d*_0_ distances (Figure 3B)—but also an additional flipped χ_2_ conformation centered around +90° (Figure 3B). This flipped ensemble, which has not been reported in *Is*PETase crystal structures, yields only sparsely populated configurations with *d*_0_ < 0.35 nm, occurring exclusively when χ_2_ approaches +40° or +90°. Because these states occur only at high-energy values, they drastically limit the population of catalytically competent configurations in the cPET complex. Overall, these results show that although Trp185 can still adopt wobbling conformations in the presence of cPET, these orientations do not lead to productive substrate positioning. The rigidity of cPET, together with the steric hindrance of Trp185, severely limits access to catalytically competent arrangements, highlighting this residue—critical for aPET binding—as key hotspots for engineering enhanced cPET degradation.

**Figure 3:**
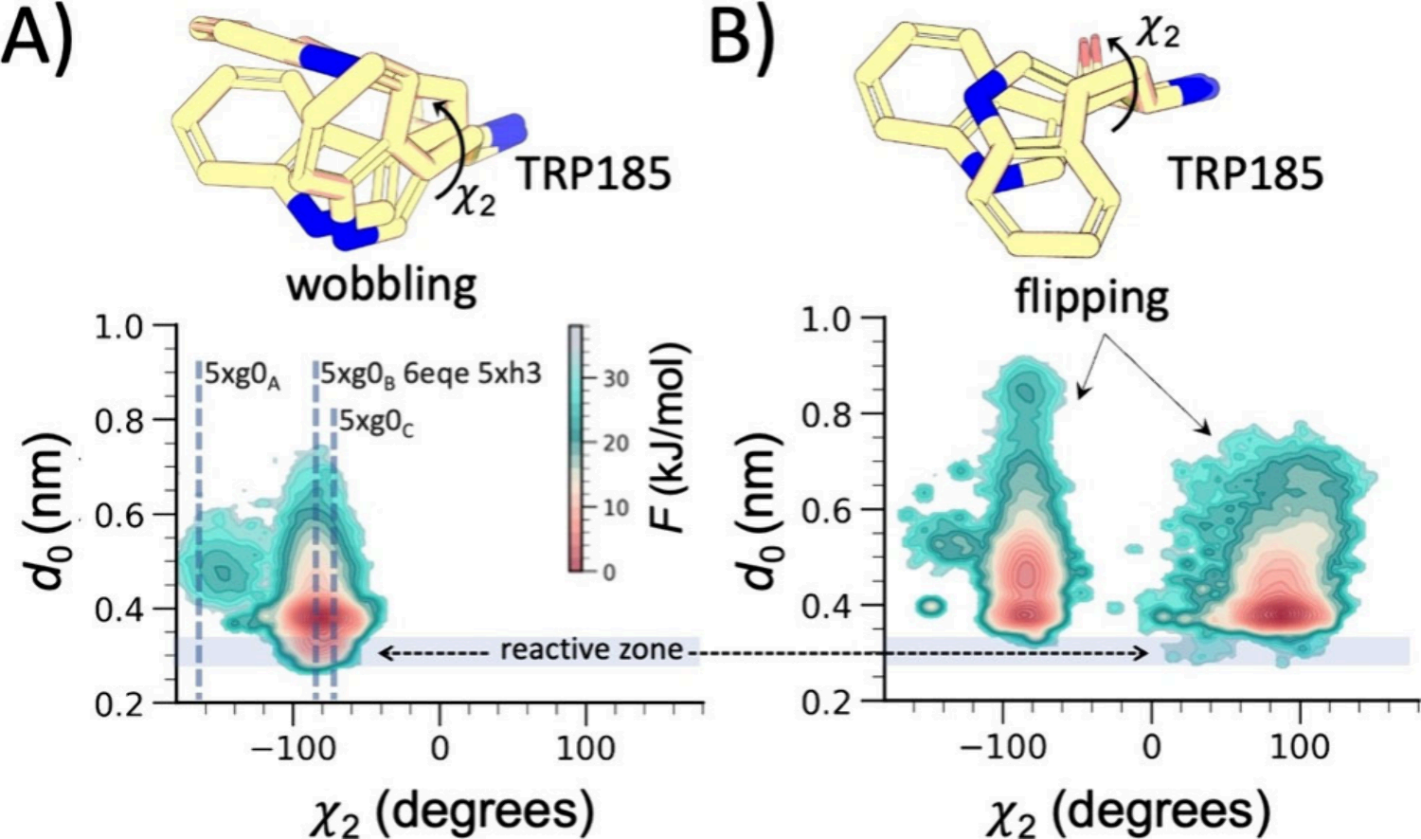
The structural origin of the energetic difference between aPET and cPET can be attributed to Trp185 conformational gating. Free-energy surfaces of *Is*PETase bound to (A) amorphous and (B) crystalline PET, shown as functions of the *d*_0_ distance and the χ_2_ torsion angle of Trp185, together with representative molecular structures illustrating the Trp185 conformational ensemble. Gray dashed vertical lines, labeled with the respective PDB codes, indicate the χ_2_ angles observed in *Is*PETase crystal structures within the explored conformational space.

## Monomer Detachment Is Energetically Demanding in Crystalline PET

To obtain a general estimate of the energy required in our idealized system for PET filaments to be extracted from the melt or surface and become accessible to the enzyme active site, we calculated the free energy associated with the detachment of two idealized filaments in both crystalline and amorphous PET (Figure 4). Indeed, the capacity of a chain segment at the surface to temporarily leave the solid structure and fit into the hydrolase active site is a key determinant of catalysis.^7^ By biasing the distance between the centers of mass (COM), *d*_COM_, of each filament using well-tempered metadynamics,^29,30^ we observed the two filaments sliding past each other, revealing free-energy wells characteristic of the intermediate states involved. In particular, during the relative displacement of an aromatic PET unit, intermediate states were observed in which the EG moiety of one filament interacts with the aromatic ring of the other filament. These intermediates alternate with π–π stacking of PET aromatic moieties, resulting in well-defined free-energy basins, especially in cPET (Figure 4A, 4B). As the system progresses along the unbinding reaction coordinate, the two oligomers eventually reach a completely unbound state, as indicated by a plateau in the free-energy profile (Figure 4A). From these plots, the binding free energy (ΔF) was calculated by integrating over all bound and unbound states (Figure 4C; see Methods). cPET filaments exhibit a ΔF of approximately −60 kJ/mol, whereas aPET filaments show a substantially weaker interaction, with ΔF around −25 kJ/mol. To compare with experimental values, we apply a standard-state correction^43^ for the 12 nm cubic simulation box (see Methods), corresponding to approximately −17 kJ/mol, giving a computed standard unbinding (ΔF°_unbind_ = −ΔF°_bind_) free energy of ΔF°_unbind_ ≈ 77 and 42 kJ/mol for cPET and aPET oligomers, respectively (∼19 kJ/mol per monomer in cPET and ∼11 kJ/mol per monomer in aPET). This value can be compared with experimental estimates of fold surface free energy (σ_e_ ≈ 0.106 J/m^2^),^44,45^ which, assuming a cross-sectional area (*A*_chain_) of PET chain ≈ 0.20 nm^2^, can be converted to an energetic cost of ∼25 kJ/mol per PET repeat unit (estimated as ΔF ≈ 2 × σ_e_ × *A*_chain_ × *N*_A_).^46,47^ Our results therefore capture the local detachment energetics of PET oligomers consistently with macroscopic measurements of lamellar crystalline PET surfaces.^44,45^

**Figure 4:**
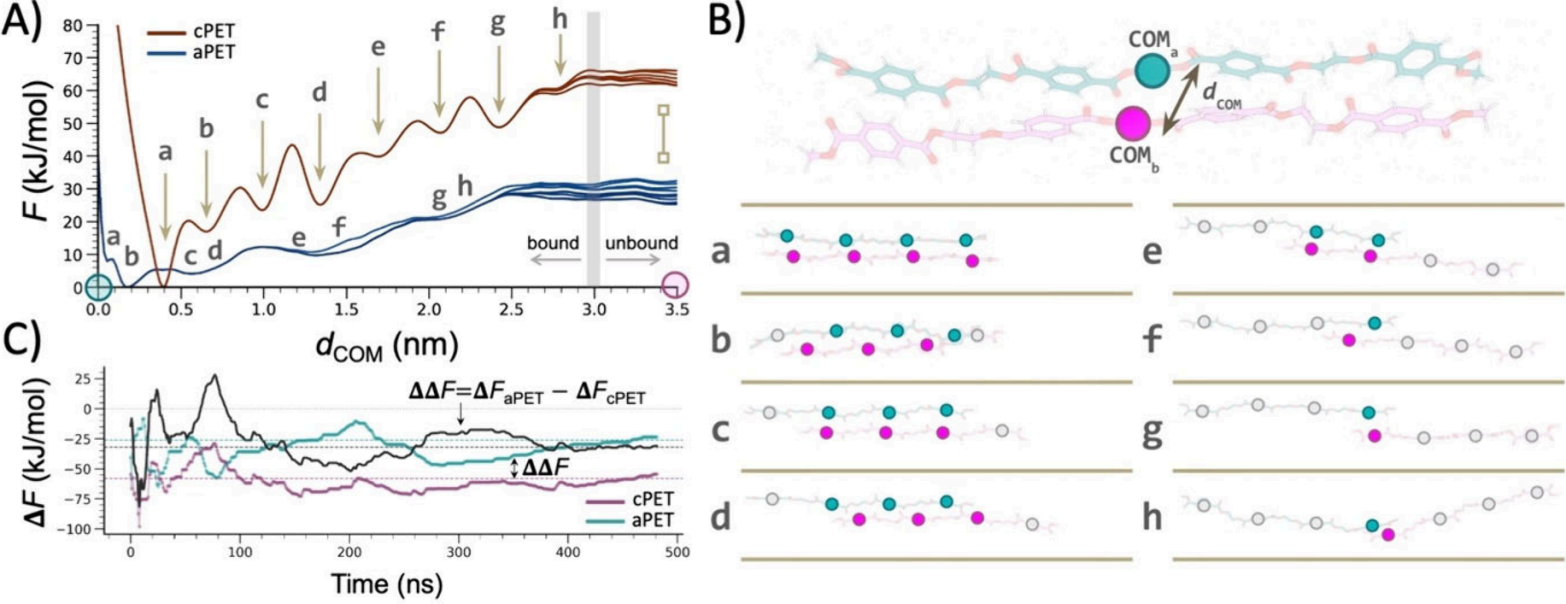
Characterization of PET chain detachment for aPET and cPET. (A) Free-energy profiles reconstructed along the distance between the centers of mass (COM), *d*_COM_, of two PET oligomers in cPET (brown) and aPET (blue); multiple lines represent the profiles extracted from the last 50 ns of simulations at 5 ns intervals (see also Supplementary Figure S5A). (B) Schematic representation of the dissociation pathway characterized by metadynamics simulations. Unbinding and rebinding events were sampled by biasing the distance between the center of mass (*d*_COM_) of the two PET oligomers. The aromatic terephthalate moieties involved in the bound state are shown in cyan and violet in the subpanels, where intermediate π–EG and π–π stacking interactions alternate before the system reaches the fully unbound configuration. Unbound moieties are depicted as gray, noncolored circles. (C) Free-energy differences, ΔF, estimated between bound and unbound states (see Methods) for cPET (violet) and aPET (cyan) as a function of the accumulated simulation time. Dashed horizontal lines indicate the average values extracted from the last 50 ns of the simulations. The relative free-energy difference, ΔΔF (black line), between aPET and cPET converges to about 35 kJ/mol after ∼400 ns.

When analyzing the free-energy difference between all bound states and the unbound states as a function of simulation time (Figure 4C), we observed that the relative ΔΔF between aPET and cPET (black line in Figure 4C) stabilizes after about 400 ns, converging to an approximate 35 kJ/mol difference. This indicates that, in our idealized system of two freely interacting oligomers, detaching two cPET chains requires about 35 kJ/mol more energy (approximate average cost of 9 kJ/mol per monomer) than detaching those in aPET, where the filaments are more loosely bound and thus more accessible to enzymatic cleavage. We note that individual ΔF estimates for aPET and cPET are inherently difficult to converge, because sampling the unbound region can continue to reveal new microstates over long time scales. Consequently, the slow upward drift in the ΔF traces reflects incomplete sampling of the unbound basin. Importantly, however, this does not prevent reliable estimation of the relative free-energy difference (ΔΔF) between aPET and cPET, which converges more rapidly and remains stable across the trajectories.

When comparing the estimated free energy cost of chain detachment with that required to reach a catalytically competent ensemble, it becomes apparent that detachment further limits the enzyme ability to access configurations poised for cleavage. Thus, interactions between polymer chains in the solid substrate could result in large activation barriers for complexation or detachment, thereby slowing the overall process even if the actual hydrolytic reaction is fast.^48^ While the observed wide basin of substrate attraction may facilitate proximity between the enzyme active site and a single PET chain (for both aPET and cPET), progression toward a catalytically competent state is significantly hindered in the case of cPET. This reinforces the broader notion that interfacial phenomena, particularly extended interactions between the enzyme and the polymer, may play a critical role in stabilizing the enzyme–substrate complex and enabling effective biocatalysis, provided that these interactions satisfy the Sabatier principle.^49^

## Discussion, Applications, and Limitations of the Model

This study provides a mechanistic framework for understanding the molecular and energetic constraints to the enzymatic depolymerization of cPET by a prototypical PETase. By integrating experimental conformational preferences of PET chains with enhanced-sampling molecular dynamics simulations, we reconstructed the free-energy landscape associated with productive substrate–enzyme catalytic ensembles. The comparison of amorphous and crystalline PET oligomers, combined with the analysis of chain detachments, provides quantitative catalytic insight into the thermodynamic penalties that limit the population of catalytically competent states, revealing the molecular determinants of enzymatic inactivity on crystalline PET.

The involvement of the conserved Trp185 in stabilizing the binding of amorphous PET on one hand, and in hindering the proximity of the reactive center of cPET to the catalytic serine on the other, underscores the critical role of conformational matching between enzyme and substrate in determining catalytic efficiency against cPET. This finding suggests that rational active-site redesign or even the development of alternative scaffolds may be required to overcome the intrinsic limitations of cPET depolymerization imposed by the active-site architectures typically found in α/β-hydrolases. It is interesting to note that among α/β-hydrolase folds, the cutinase from *Humicola insolens* (HiC)^50^ lacks the aromatic or bulky residue found at the position equivalent to Trp185 in *Is*PETase (Figure 5). Consequently, its active site adopts a straighter and open architecture, potentially allowing direct access from the catalytic serine to the reactive centers of linear PET chains, without the steric “bumps” that are typical of PET hydrolases. Intriguingly, HiC has been reported to exhibit activity against ball-milled, high-crystallinity PET under moist-solid reaction–mixture conditions.^17^ It should be acknowledged that ball milling at room temperature induces partial amorphization of the polyester while simultaneously increasing its surface area and roughness, thereby enhancing the efficiency of enzymatic hydrolysis.^51^ The computational procedure presented here, together with future developments in synergy with reaction-mechanism quantum mechanical calculations, may enable quantitative characterization and shed light on the energetics underlying cPET reactivity with HiC and other α/β-hydrolases with peculiar structural features,^52^ possibly reconciling the relationship between conformational equilibria, reactivity, and catalytic activity.

**Figure 5:**
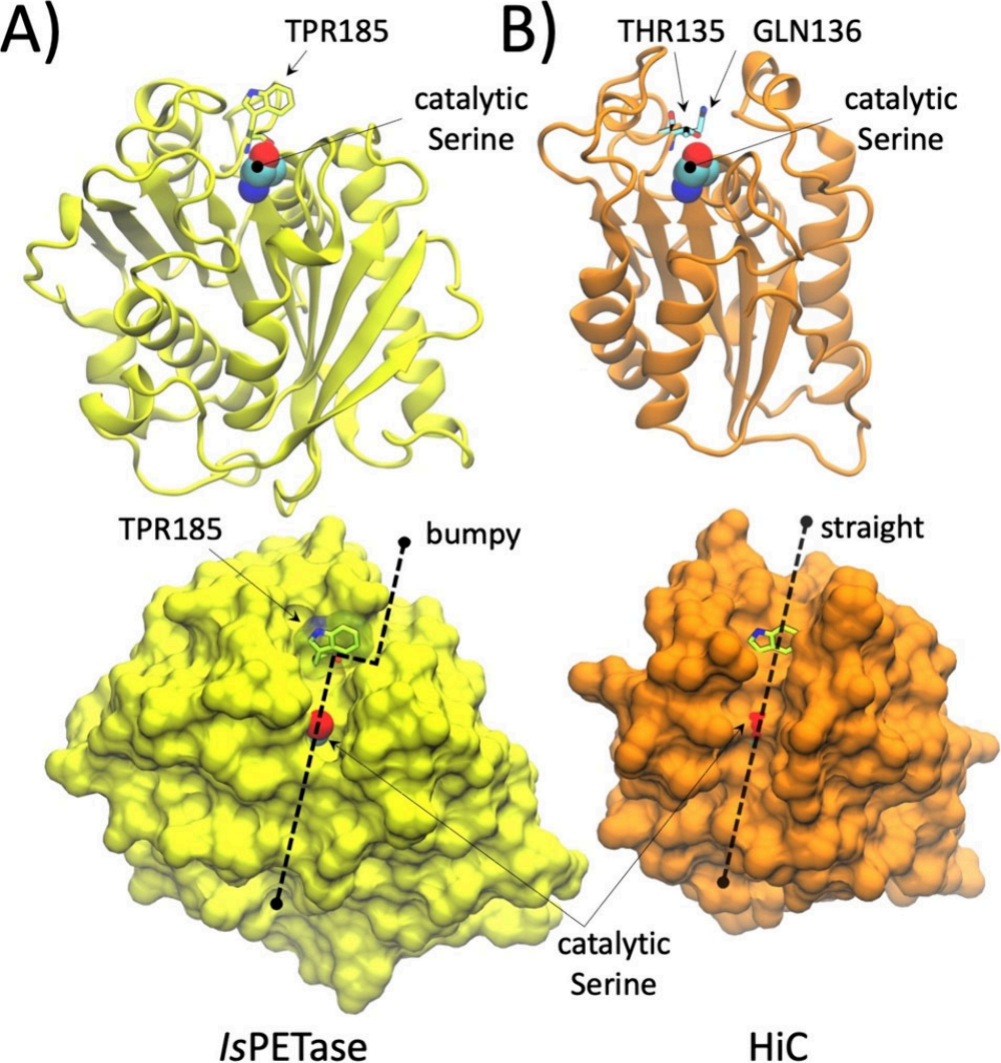
Structural features of α/β-hydrolase folds that may facilitate or restrict access to cPET. (A) 3D representation of the overall fold of *Is*PETase, in which the conserved Trp185 in subsite I facilitates interaction with amorphous PET chains but hinders access to cPET; Trp185 creates bumps on the molecular surface surrounding the active site (lower panel). (B) The cutinase HiC is smaller than other PETases and cutinases and lacks the aromatic or bulky residue at the position equivalent to Trp185 in *Is*PETase, which corresponds to Thr135 and Gln136 in HiC (following the numbering of PDB 4OYY).^50^ Its active site forms a straight binding pocket compatible with cPET chains (lower panel).

Several limitations should be acknowledged. First, we modeled short PET oligomers and used idealized representations of crystalline and amorphous states. While essential for reducing system complexity and isolating key energetic contributions, these simplifications do not fully capture the structural and morphological heterogeneity of PET matrices. In addition, the simulations do not describe interfacial dynamics or surface-binding effects that likely modulate molecular behavior in heterogeneous environments. The oligomer (un)­binding simulations presented here should be viewed as indicators of the relative ease with which aPET versus cPET chains can disengage from their local environments, rather than as a full representation of polymer-surface energetics. This limitation arises because the simulations do not include an explicit polymer matrix^9,22^ and rely on a fixed-charge force field such as GAFF,^53^ which provides only an approximate description of π–π interactions, although recent work has encouragingly shown the suitability of using the GAFF force field for studying plastic polymeric systems.^9^ Caution should also be applied when directly comparing these PET–PET dimerization free energies with those associated with catalytic-ensemble formation in the enzyme, as such force-field limitations may affect the absolute magnitude of PET–PET interactions to a greater extent than in the PET–enzyme systems. While our results are based on a single enzyme, we anticipate comparable energetic trends across PETase variants, particularly those sharing the conserved role of Trp185 dynamics.^54–57^ The systematic application of the approach presented here may serve as a powerful tool to probe structure–function relationships and to assess the conformational complementarity between cPET and the active sites of prospective biocatalysts. In synergy with quantum mechanical calculations, this may reveal the intrinsic capability of the enzyme to efficiently react with cPET substrates. Overall, the computational strategy introduced here provides a framework for probing how enzyme active-site architectures interact with substrates that display both crystalline and amorphous conformational states. We hope this work facilitates the integration of sustainability considerations, such as the ability to depolymerize cPET with reduced reliance on costly amorphization processes, directly into enzyme design and discovery through the characterization of the molecular determinants underlying PET depolymerization.

## Methods

### Molecular Models and Simulation Setups

The crystal structure of *Is*PETase (PDB entry 6EQE)^58^ was used as a reference to model the complex with a PET oligomer of four subunits (PET_4_). The structure of PET_4_ was generated using Maestro (Schrödinger, LCC, New York, NY, USA). The oligomer was modeled with the Generalized Amber Force Field (GAFF)^53^ and partial atomic charges were assigned to the extended conformation^59^ using the Restrained Electrostatic Potential fit^60^ method at the HF/6–31G* level of theory using Gaussian16.B01 (Gaussian, Inc. Wallingford, CT, USA). Parameters and topology files were prepared with Acpype.^61^ We generated the initial configuration of the *Is*PETase–PET_4_ complex using a pulling-MD protocol^18^ (see Figure S1 and the SI for more details) that gradually aligned one of the two central terephthalic acid units of a randomly positioned PET_4_ molecule with the monomeric 2-hydroxyethyl methyl terephthalate (HEMT) ligand in the 5XH3 crystal structure,^41^ by reducing the RMSD between the PET_4_ central unit and the reference HEMT configuration to 0 nm. We note that while this procedure enables the smooth insertion of the PET oligomer into the active site, consistent with the crystallographic binding mode of the model substrate, it was not intended to study the binding process itself. Instead, it was used to generate an initial “guess” configuration of the *Is*PETase–PET_4_ complexes. The *Is*PETase–PET_4_ systems were solvated with a 1 nm-thick truncated octahedron box of TIP3P^62^ water molecules with periodic boundary conditions and neutralized with an excess NaCl concentration of 0.15 M. After minimization and thermalization in the canonical ensemble (NVT), each system was equilibrated at constant pressure and temperature (1 atm, 25 °C) using the stochastic velocity rescaling thermostat^63^ and the Parrinello–Rahman barostat,^64^ while smoothly releasing the initial restraints on the RMSD distance with respect to the template monomeric substrate within 100 ns. The resulting system was further evolved for a cumulative time of 100 ns and then used as a starting point for the combined HREX-metadynamics simulations that explored the energetics of the PET/enzyme system in proximity of the catalytic ensemble. Equations of motion were integrated with a time step of 2 fs. For all nonbonded interactions, the real space cutoff was set to 1.2 nm, and the electrostatic long-range interactions were treated using the default particle-mesh Ewald settings.^65^ Bonds involving hydrogen atoms were constrained using the LINCS algorithm.^66^ All MD simulations were carried out with GROMACS 2021.4 ^67^ patched with PLUMED 2.8 ^34,68,69^ using the Amber ff99SB-ILDN force field.^70^ 3D structures were visualized with VMD.^71^ GROMACS and PLUMED^34,68,72^ input files are available at PLUMED-NEST^69^ (plumID:25.029).

### Modeling Crystalline and Amorphous PET States

One PET_4_ oligomer was embedded into 1 nm-thick truncated octahedron box of TIP3P^62^ water molecules with periodic boundary conditions. After thermalization and equilibration in the NPT ensemble at 1 atm, 300 K, we controlled the OC–CO ethylene-glycol torsion Ψ (Figure 1) via distance-based restraints applied at the terminal carbonyl carbons of PET_4_ with values of *d*_1_ ranging from 0.1 to 4.0 nm (Figure 1), using PLUMED lower-wall potentials (k = 1500 kJ mol^–1^ nm^–2^) during independent 100 ns long plain MD runs. Specifically, when the minimum distance (*d*_1_) between the terminal C=O groups of subunits 1 and 4 in PET_4_ was limited to 2.4 and 4.0 nm, the oligomer reproduced the conformational behavior characteristic of longer polymeric substrates in the amorphous and crystalline states, respectively. *Gauche* conformations were defined as those with Ψ between −120° and 120°, while *trans* conformations were defined as Ψ greater than 120° or less than −120°. This resulted in a *trans*-to-*gauche* ratio of Ψ comparable to the 1:9 *t*/*g* ratio observed in amorphous PET,^10^ and a fully *trans* conformation consistent with crystalline PET.^11,12^

### Detachment Simulations in Crystalline and Amorphous PET Chains

Two PET_4_ oligomers were embedded into 12 nm-thick TIP3P water box (reaching a total of 146976 atoms), while controlling the distance *d*_1_ of each oligomer so that to model the interaction between two amorphous (*d*_1_ = 2.4 nm) and crystalline (*d*_1_ = 4.0 nm) PET chains. A large solvated water box was used to allow full sliding and detachment of the PET oligomers, particularly cPET, while avoiding boundary artifacts. After thermalization and equilibration in the NPT ensemble, unbinding and rebinding events of the PET chains were sampled using well-tempered metadynamics^29,30^ by biasing the distance between the center of mass (*d*_COM_) of the two PET oligomers (exemplified also in Figure 4). Each aPET and cPET system was run for 500 ns of wt-MTD simulations, using initial Gaussian heights of 0.5 kJ mol^–1^, a width of 0.05 nm, a bias factor of 15, and an addition frequency of 1 ps. To estimate the ΔF values shown in Figure 4C, the bound and unbound states were defined as those with *d*_COM_ values lower and higher than 3 nm, respectively. For each state, the free-energy contribution was obtained by integrating the Boltzmann-weighted populations over the corresponding region of the reaction coordinate.^72^

### Combined HREX-wt-MTD Simulations

Replica exchange methods simulate multiple replicas of the same system in parallel, each evolving on a modified energy surface, typically at different temperatures or effective temperatures. A “cold” (unbiased) replica provides the target statistics, while “hot” replicas accelerate sampling by overcoming energy barriers. Intermediate replicas enable smooth transitions between ensembles. By periodically swapping configurations via Monte Carlo moves, the system explores different free-energy landscapes and efficiently samples multiple conformational states.^31–34^ The HREX settings^31,32^ used here, in particular, modify the free-energy surfaces by modulating only the force-field terms contributing to energy barriers (charge; epsilon Lennard-Jones parameter; proper dihedral).^34^ We used eight replicas and we scaled the force field parameters of all the atoms of PET_4_ (thus resulting in the Replica Exchange with Solute Tempering, REST2, formulation)^32,33^ with values of λ ranging from 1 to 0.43 following a geometric distribution (1, 0.885511, 0.784132, 0.694357, 0.614861, 0.544467, 0.482133, 0.426934). Exchanges were attempted every 500 steps (1 ps). The acceptance rate ranged from 39% to 48%. Each replica ran for 800 ns with a cumulative sampling time of 6.4 μs per system. Concurrently with the enhanced-sampling provided by the HREX setup described above, a wt-MTD bias^29,30^ was introduced to maximize sampling of catalytic ensembles (Supplementary Figures S2 and S4).^73^ The free energy of the system was “filled” iteratively by a sum of repulsive Gaussians positioned along a suitably chosen set of collective variables (CVs), thereby enhancing the exploration of different minima in the selected CV space. In this framework, the distances between the carbonyl oxygen of PET_4_ and the backbone NH groups of M161 and Y87 (labeled in Figure 3 as *h*_0_ and *h*_1_, respectively) were used as CVs to bias the exploration of the configurational space with a bias factor of 15. The free-energy landscape was projected onto CVs different from the biased ones through reweighting.^74^ Systematic error was estimated by block analysis (Figure S2). To optimize the efficiency of sampling, *h*_0_ and *h*_1_ were limited to a maximum value of 0.6 nm by a wall potential with k = 5000 kJ mol^–1^ nm^–2^. The initial height, *w*, of the Gaussians was set to 0.5 kJ mol^–1^ and the addition frequency to 1 ps. To improve filling the energetic minima in the “hot” replicas with λ < 1 (high effective temperature in the HREX-ladder), the height of the Gaussians in the corresponding replicas was scaled as *w*_*s*_ = *w*/λ.^75^ The width of the Gaussians was set to 0.05 nm for all systems. All the analysis and statistics were collected from the unscaled (λ = 1; *w* = 0.5 kJ mol^–1^) replica.

## Supplementary Material





## Data Availability

GROMACS and PLUMED input files are available on the PLUMED-NEST^69^ (https://www.plumed-nest.org/) with plumID:25.029.

## Notes

Additional computational details and methods, including additional computational setup and free-energy projections, along with several CV combinations (PDF)
Transparent Peer Review report available (PDF)

## References

[1] Cottom J. W., Cook E., Velis C. A. (2024). A Local-to-Global
Emissions Inventory
of Macroplastic Pollution. Nature.

[2] Pottinger A. S., Geyer R., Biyani N., Martinez C. C., Nathan N., Morse M. R., Liu C., Hu S., de Bruyn M., Boettiger C., Baker E., McCauley D. J. (2024). Pathways to Reduce
Global Plastic Waste Mismanagement and Greenhouse Gas Emissions by
2050. Science (80-.)..

[3] Zimmermann W. (2025). Polyester-Degrading
Enzymes in a Circular Economy of Plastics. Nat.
Rev. Bioeng..

[4] Zimmermann W. (2025). Biocatalytic
Recycling of Plastics: Facts and Fiction. Chem.
Sci..

[5] Tournier V., Duquesne S., Guillamot F., Cramail H., Taton D., Marty A., André I. (2023). Enzymes’ Power for Plastics
Degradation. Chem. Rev..

[6] Colizzi F., Blázquez-Sánchez P., Bussi G., André I., Ballabio F., Bayer T., Bertocchini F., Butenschön E., Carosati E., Piero A. De, Pede-Mattatelli A. Di, Faggian L., Favaro L., Fernandes P. A., Ferretti A., Fojan P., Gagsteiger A., García-Ruiz E., Gardossi L., Giorgino T., Graefe N., Gunkel J., Jiménez D. J., Keller M. B., Künze G., Lim S., Lippens G., Maria-Solano M. A., Mitusińska K., Moliner V., Molla G., Myburgh M. W., Panel N., Ramírez-Sarmiento C. A., Rocher D., Strodel B., Ruiz B. T., Turak O., Varrone C., Vezzini D., Westh P., Zimmermann W. (2025). Computations Meet Experiments to
Advance the Enzymatic Depolymerization of Plastics One Atom at a Time. arXiv Prepr..

[7] Marten E., Müller R. J., Deckwer W. D. (2005). Studies on the Enzymatic
Hydrolysis
of Polyesters. II. Aliphatic-Aromatic Copolyesters. Polym. Degrad. Stab..

[8] Møller M. S., Bleckert A., Jäckering A., Strodel B. (2025). Unraveling the Relationship
between PET Surfaces and Their Hydrolases. Trends
Biochem. Sci..

[9] Paiva P., Ippoliti E., Carloni P., Fernandes P. A., Ramos M. J. (2025). Atomistic Adsorption of PETase onto
Large-Scale PET
3D-Models That Mimic Reality. Phys. Chem. Chem.
Phys..

[10] Wei R., Song C., Gräsing D., Schneider T., Bielytskyi P., Böttcher D., Matysik J., Bornscheuer U. T., Zimmermann W. (2019). Conformational Fitting of a Flexible Oligomeric Substrate
Does Not Explain the Enzymatic PET Degradation. Nature Communications..

[11] Schmidt-Rohr K., Hu W., Zumbulyadis N. (1998). Elucidation
of the Chain Conformation in a Glassy Polyester,
PET, by Two-Dimensional NMR. Science (80-.)..

[12] Schmidt-Rohr, K. ; Spiess, H. W. Multidimensional Solid-State NMR and Polymers; Academic Press, London, 1994. 10.1016/C2009-0-21335-3.

[13] Lopez-Lorenzo X., Ranjani G., Syrén P.-O. (2025). Front Cover: Conformational Selection
in Enzyme-Catalyzed Depolymerization of Bio-Based Polyesters (ChemBioChem
2/2025). ChemBioChem..

[14] Guo B., Vanga S. R., Lopez-Lorenzo X., Saenz-Mendez P., Ericsson S. R., Fang Y., Ye X., Schriever K., Bäckström E., Biundo A., Zubarev R. A., Furó I., Hakkarainen M., Syrén P. O. (2022). Conformational
Selection in Biocatalytic Plastic Degradation by PETase. ACS Catal..

[15] Chen Z., Duan R., Xiao Y., Wei Y., Zhang H., Sun X., Wang S., Cheng Y., Wang X., Tong S., Yao Y., Zhu C., Yang H., Wang Y., Wang Z. (2022). Biodegradation
of Highly Crystallized Poly­(Ethylene Terephthalate) through Cell Surface
Codisplay of Bacterial PETase and Hydrophobin. Nat. Commun..

[16] Norton-Baker B., Komp E., Gado J. E., Denton M. C. R., Mathews I. I., Murphy N. P., Erickson E., Storment O. O., Sarangi R., Gauthier N. P., McGeehan J. E., Beckham G. T. (2025). Machine Learning-Guided
Identification of PET Hydrolases from Natural Diversity. ACS Catal..

[17] Kaabel S., Daniel Therien J. P., Deschênes C. E., Duncan D., Friščic T., Auclair K. (2021). Enzymatic Depolymerization of Highly Crystalline Polyethylene
Terephthalate Enabled in Moist-Solid Reaction Mixtures. Proc. Natl. Acad. Sci. U.S.A..

[18] Falkenstein P., Zhao Z., Di Pede-Mattatelli A., Künze G., Sommer M., Sonnendecker C., Zimmermann W., Colizzi F., Matysik J., Song C. (2023). On the Binding
Mode
and Molecular Mechanism of Enzymatic Polyethylene Terephthalate Degradation. ACS Catal..

[19] Falkenstein, P. ; Wei, R. ; Matysik, J. ; Song, C. Mechanistic Investigation of Enzymatic Degradation of Polyethylene Terephthalate by Nuclear Magnetic Resonance. Methods in Enzymology; Elsevier, 2021; Vol. 648, pp 231–252. 10.1016/bs.mie.2020.11.002. 33579405

[20] Charlier C., Gavalda S., Grga J., Perrot L., Gabrielli V., Löhr F., Schörghuber J., Lichtenecker R., Arnal G., Marty A., Tournier V., Lippens G. (2024). Exploring
the PH Dependence of an Improved PETase. Biophys.
J..

[21] Charlier C., Gavalda S., Borsenberger V., Duquesne S., Marty A., Tournier V., Lippens G. (2022). An NMR Look
at an Engineered PET
Depolymerase. Biophys. J..

[22] Jäckering A., Göttsch F., Schäffler M., Doerr M., Bornscheuer U. T., Wei R., Strodel B. (2024). From Bulk to Binding: Decoding the Entry of PET into
Hydrolase Binding Pockets. JACS Au.

[23] Sahihi M., Fayon P., Nauton L., Goujon F., Devémy J., Dequidt A., Hauret P., Malfreyt P. (2024). Probing Enzymatic PET
Degradation: Molecular Dynamics Analysis of Cutinase Adsorption and
Stability. J. Chem. Inf. Model..

[24] Chen S., Akram E., Liang H., Qiao W., Zhang Y., Haider S., Cao Y. (2025). Insight into How PETase Functions
at the Solid-Liquid Interface and an Activity-Stability Trade-Off. Angew. Chemie - Int. Ed..

[25] Zheng M., Li Y., Dong W., Zhang Q., Wang W. (2024). Hydrolase-Catalyzed
Depolymerization Mechanism toward Crystalline and Amorphous Polyethylene
Terephthalate. ACS Sustain. Chem. Eng..

[26] Lazarenko D., Schmidt G. P., Crowley M. F., Beckham G. T., Knott B. C. (2025). Molecular
Details of Polyester Decrystallization via Molecular Simulation. Macromolecules.

[27] Yoshida S., Hiraga K., Takehana T., Taniguchi I., Yamaji H., Maeda Y., Toyohara K., Miyamoto K., Kimura Y., Oda K. (2016). A Bacterium That Degrades
and Assimilates
Poly­(Ethylene Terephthalate). Science (80-.)..

[28] Wallace N. E., Adams M. C., Chafin A. C., Jones D. D., Tsui C. L., Gruber T. D. (2020). The Highly Crystalline PET Found in Plastic Water Bottles
Does Not Support the Growth of the PETase-Producing Bacterium Ideonella
Sakaiensis. Environ. Microbiol. Rep..

[29] Barducci A., Bonomi M., Parrinello M. (2011). Metadynamics. Wiley Interdiscip. Rev. Comput. Mol. Sci..

[30] Barducci A., Bussi G., Parrinello M. (2008). Well-Tempered
Metadynamics: A Smoothly-Converging
and Tunable Free-Energy Method. Phys. Rev. Lett..

[31] Sugita Y., Okamoto Y. (1999). Replica-Exchange Molecular
Dynamics Method for Protein
Folding. Chem. Phys. Lett..

[32] Wang L., Friesner R. A., Berne B. J. (2011). Replica
Exchange with Solute Scaling:
A More Efficient Version of Replica Exchange with Solute Tempering
(REST2). J. Phys. Chem. B.

[33] Colizzi F. (2025). Leveraging
Cryptic Ligand Envelopes through Enhanced Molecular Simulations. J. Phys. Chem. Lett..

[34] Bussi G. (2014). Hamiltonian
Replica Exchange in GROMACS: A Flexible Implementation. Mol. Phys..

[35] Jerves C., Neves R. P. P., Ramos M. J., da Silva S., Fernandes P. A. (2021). Reaction
Mechanism of the PET Degrading Enzyme PETase Studied with DFT/MM Molecular
Dynamics Simulations. ACS Catal..

[36] García-Meseguer R., Ortí E., Tuñón I., Ruiz-Pernía J. J., Aragó J. (2023). Insights into the Enhancement of the Poly­(Ethylene
Terephthalate) Degradation by FAST-PETase from Computational Modeling. J. Am. Chem. Soc..

[37] Burgin T., Pollard B. C., Knott B. C., Mayes H. B., Crowley M. F., McGeehan J. E., Beckham G. T., Woodcock H. L. (2024). The Reaction Mechanism
of the Ideonella Sakaiensis PETase Enzyme. Commun.
Chem..

[38] Boneta S., Arafet K., Moliner V. (2021). QM/MM Study
of the Enzymatic Biodegradation
Mechanism of Polyethylene Terephthalate. J.
Chem. Inf. Model..

[39] Maria-Solano M. A., Serrano-Hervás E., Romero-Rivera A., Iglesias-Fernández J., Osuna S. (2018). Role of Conformational
Dynamics in the Evolution of Novel Enzyme Function. Chem. Commun..

[40] Petrović D., Frank D., Kamerlin S. C. L., Hoffmann K., Strodel B. (2017). Shuffling
Active Site Substate Populations Affects Catalytic Activity: The Case
of Glucose Oxidase. ACS Catal..

[41] Han X., Liu W., Huang J.-W., Ma J., Zheng Y., Ko T.-P., Xu L., Cheng Y.-S., Chen C.-C., Guo R.-T. (2017). Structural Insight
into Catalytic Mechanism of PET Hydrolase. Nat.
Commun..

[42] Chen C. C., Han X., Li X., Jiang P., Niu D., Ma L., Liu W., Li S., Qu Y., Hu H., Min J., Yang Y., Zhang L., Zeng W., Huang J. W., Dai L., Guo R. T. (2021). General Features to Enhance Enzymatic Activity of Poly­(Ethylene
Terephthalate) Hydrolysis. Nat. Catal..

[43] General I. J. (2010). A Note
on the Standard State’s Binding Free Energy. J. Chem. Theory Comput..

[44] Tang R., Liggat J. J., Siew W. H. (2018). Partial
Discharge Behaviour of Biaxially
Orientated PET Films: The Effect of Crystalline Morphology. Polym. Degrad. Stab..

[45] Lu X. F., Hay J. N. (2001). Isothermal Crystallization Kinetics and Melting Behaviour
of Poly­(Ethylene Terephthalate). Polymer (Guildf)..

[46] Toda A. (2025). Melting Kinetics
of Polymer Crystals. Polymer Journal..

[47] Zhang R. C., Sun D., Lu A., Zhong M., Xiong G., Wan Y. (2017). Equilibrium
Melting Temperature of Polymorphic Poly­(L-Lactide) and Its Supercooling
Dependence on Growth Kinetics. Polymers (Basel)..

[48] Bååth J. A., Borch K., Jensen K., Brask J., Westh P. (2021). Comparative
Biochemistry of Four Polyester (PET) Hydrolases**. ChemBioChem..

[49] Arnling
Bååth J., Jensen K., Borch K., Westh P., Kari J. (2022). Sabatier Principle for Rationalizing Enzymatic Hydrolysis of a Synthetic
Polyester. JACS Au.

[50] Kold D., Dauter Z., Laustsen A. K., Brzozowski A. M., Turkenburg J. P., Nielsen A. D., Koldsø H., Petersen E., Schiøtt B., De Maria L., Wilson K. S., Svendsen A., Wimmer R. (2014). Thermodynamic and Structural Investigation
of the Specific SDS Binding of Humicola Insolens Cutinase. Protein Sci..

[51] Zhou Y., Zhang J., Zheng Y., Lin W., You S., Wang M., Su R., Qi W. (2025). Simple Enzymatic
Depolymerization
Process Based on Rapid Ball Milling Pretreatment for High-Crystalline
Polyethylene Terephthalate Fibers. Bioresour.
Technol..

[52] Turak O., Gagsteiger A., Upadhyay A., Kriegel M., Salein P., Böhnke-Brandt S., Agarwal S., Borchert E., Höcker B. (2025). A Third Type
of PETase from the Marine *Halopseudomonas* Lineage. Protein Sci..

[53] Wang J., Wolf R. M., Caldwell J. W., Kollman P. A., Case D. A. (2004). Development
and Testing of a General Amber Force Field. J. Comput. Chem..

[54] Jäckering A., van der Kamp M., Strodel B., Zinovjev K. (2024). Influence of Wobbling
Tryptophan and Mutations on PET Degradation Explored by QM/MM Free
Energy Calculations. J. Chem. Inf. Model..

[55] Crnjar A., Griñen A., Kamerlin S. C. L., Ramírez-Sarmiento C. A. (2023). Conformational
Selection of a Tryptophan Side Chain Drives the Generalized Increase
in Activity of PET Hydrolases through a Ser/Ile Double Mutation. ACS Org. Inorg. Au.

[56] Chen C. C., Han X., Li X., Jiang P., Niu D., Ma L., Liu W., Li S., Qu Y., Hu H., Min J., Yang Y., Zhang L., Zeng W., Huang J. W., Dai L., Guo R. T. (2021). General Features to Enhance Enzymatic Activity of Poly­(Ethylene
Terephthalate) Hydrolysis. Nat. Catal..

[57] Chen C. C., Han X., Ko T. P., Liu W., Guo R. T. (2018). Structural Studies
Reveal the Molecular Mechanism of PETase. FEBS
Journal..

[58] Austin H. P., Allen M. D., Donohoe B. S., Rorrer N. A., Kearns F. L., Silveira R. L., Pollard B. C., Dominick G., Duman R., Omari K. El, Mykhaylyk V., Wagner A., Michener W. E., Amore A., Skaf M. S., Crowley M. F., Thorne A. W., Johnson C. W., Lee Woodcock H., McGeehan J. E., Beckham G. T. (2018). Characterization
and Engineering of a Plastic-Degrading Aromatic Polyesterase. Proc. Natl. Acad. Sci. U.S.A..

[59] Colizzi F., Hospital A., Zivanovic S., Orozco M. (2019). Predicting the Limit
of Intramolecular Hydrogen Bonding with Classical Molecular Dynamics. Angew. Chemie - Int. Ed..

[60] Bayly C. I., Cieplak P., Cornell W. D., Kollman P. A. (1993). A Well-Behaved Electrostatic
Potential Based Method Using Charge Restraints for Deriving Atomic
Charges: The RESP Model. J. Phys. Chem..

[61] Sousa
Da Silva A. W., Vranken W. F. (2012). ACPYPE - AnteChamber PYthon Parser
InterfacE. BMC Res. Notes.

[62] Jorgensen W. L. (1981). Transferable
Intermolecular Potential Functions for Water, Alcohols, and Ethers.
Application to Liquid Water. J. Am. Chem. Soc..

[63] Bussi G., Donadio D., Parrinello M. (2007). Canonical Sampling through Velocity
Rescaling. J. Chem. Phys..

[64] Parrinello M., Rahman A. (1981). Polymorphic Transitions
in Single Crystals: A New Molecular
Dynamics Method. J. Appl. Phys..

[65] Darden T., York D., Pedersen L. (1993). Particle Mesh
Ewald: An N·log­(N)
Method for Ewald Sums in Large Systems. J. Chem.
Phys..

[66] Hess B. (2008). P-LINCS: A
Parallel Linear Constraint Solver for Molecular Simulation. J. Chem. Theory Comput..

[67] Hess B., Kutzner C., Van Der
Spoel D., Lindahl E. (2008). GRGMACS 4: Algorithms
for Highly Efficient, Load-Balanced, and Scalable Molecular Simulation. J. Chem. Theory Comput..

[68] Tribello G. A., Bonomi M., Branduardi D., Camilloni C., Bussi G. (2014). PLUMED 2: New Feathers for an Old
Bird. Comput.
Phys. Commun..

[69] Bonomi M., Bussi G., Camilloni C., Tribello G. A., Banáš P., Barducci A., Bernetti M., Bolhuis P. G., Bottaro S., Branduardi D., Capelli R., Carloni P., Ceriotti M., Cesari A., Chen H., Chen W., Colizzi F., De S., De La Pierre M., Donadio D., Drobot V., Ensing B., Ferguson A. L., Filizola M., Fraser J. S., Fu H., Gasparotto P., Gervasio F. L., Giberti F., Gil-Ley A., Giorgino T., Heller G. T., Hocky G. M., Iannuzzi M., Invernizzi M., Jelfs K. E., Jussupow A., Kirilin E., Laio A., Limongelli V., Lindorff-Larsen K., Löhr T., Marinelli F., Martin-Samos L., Masetti M., Meyer R., Michaelides A., Molteni C., Morishita T., Nava M., Paissoni C., Papaleo E., Parrinello M., Pfaendtner J., Piaggi P., Piccini G. M., Pietropaolo A., Pietrucci F., Pipolo S., Provasi D., Quigley D., Raiteri P., Raniolo S., Rydzewski J., Salvalaglio M., Sosso G. C., Spiwok V., Šponer J., Swenson D. W. H., Tiwary P., Valsson O., Vendruscolo M., Voth G. A., White A. (2019). Promoting Transparency and Reproducibility
in Enhanced Molecular Simulations. Nat. Methods.

[70] Lindorff-Larsen K., Piana S., Palmo K., Maragakis P., Klepeis J. L., Dror R. O., Shaw D. E. (2010). Improved Side-chain
Torsion Potentials for the Amber Ff99SB Protein Force Field. Proteins Struct. Funct. Bioinforma..

[71] Humphrey W., Dalke A., Schulten K. (1996). VMD: Visual
Molecular Dynamics. J. Mol. Graph..

[72] Tribello G. A., Bonomi M., Bussi G., Camilloni C., Armstrong B. I., Arsiccio A., Aureli S., Ballabio F., Bernetti M., Bonati L., Brookes S. G. H., Brotzakis Z. F., Capelli R., Ceriotti M., Chan K.-T., Cossio P., Dasetty S., Donadio D., Ensing B., Ferguson A. L., Fraux G., Gale J. D., Gervasio F. L., Giorgino T., Herringer N. S. M., Hocky G. M., Hoff S. E., Invernizzi M., Languin-Cattoën O., Leone V., Limongelli V., Lopez-Acevedo O., Marinelli F., Febrer Martinez P., Masetti M., Mehdi S., Michaelides A., Murtada M. H., Parrinello M., Piaggi P. M., Pietropaolo A., Pietrucci F., Pipolo S., Pritchard C., Raiteri P., Raniolo S., Rapetti D., Rizzi V., Rydzewski J., Salvalaglio M., Schran C., Seal A., Shayesteh Zadeh A., Silva T. F. D., Spiwok V., Stirnemann G., Sucerquia D., Tiwary P., Valsson O., Vendruscolo M., Voth G. A., White A. D., Wu J. (2025). PLUMED Tutorials: A
Collaborative, Community-Driven Learning Ecosystem. J. Chem. Phys..

[73] Bussi G., Laio A. (2020). Using Metadynamics to Explore Complex Free-Energy Landscapes. Nat. Rev. Phys..

[74] Branduardi D., Bussi G., Parrinello M. (2012). Metadynamics
with Adaptive Gaussians. J. Chem. Theory Comput..

[75] Bussi G., Gervasio F. L., Laio A., Parrinello M. (2006). Free-Energy
Landscape for β Hairpin Folding from Combined Parallel Tempering
and Metadynamics. J. Am. Chem. Soc..

