# Supplementary material for: Why Do PETases Struggle with Crystalline PET? Catalytic Ensemble Sampling Reveals Molecular Bottlenecks
Source: J Phys Chem Lett. 2026 Apr 16;17(17):5069–78. doi: 10.1021/acs.jpclett.6c00308 (PMC13137247; doi:10.1021/acs.jpclett.6c00308)
Supplement: Supplementary file 1 [file jz6c00308_si_001.pdf]

Supporting Information

Why Do PETases Struggle with Crystalline PET?

Catalytic Ensemble Sampling Reveals Molecular Bottlenecks

Ania Di Pede-Mattatelli[2,3], Miguel A. Maria-Solano[2], Oleksandr Haisha[2], Francesco Colizzi[1,2]*

(1) Molecular Ocean Lab, Institute for Advanced Chemistry of Catalonia, IQAC−CSIC, Carrer de Jordi Girona 18-26, 08034 Barcelona, Spain

(2) Molecular Ocean Lab, Institute of Marine Sciences, ICM−CSIC, Passeig Marítim de la Barceloneta 37-49, 08003 Barcelona, Spain

(3) Graduate Programme in Biotechnology Universitat de Barcelona, Barcelona, Spain

Correspondance: colizzi@csic.es

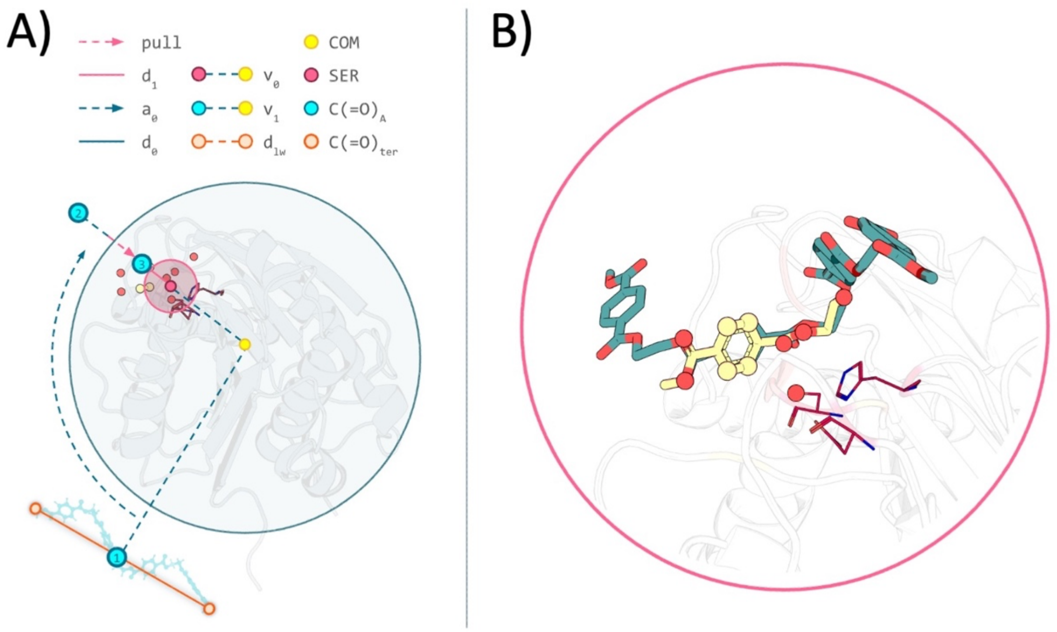

Supplementary Figure S1. Schematics of the geometrical parameters used in the steered-MD protocol to generate a starting configuration for the *Is*PETase–(PET)$_4$ complex. (A) The vector v_0 connects the center of mass (COM, yellow circle) of *Is*PETase (light blue ribbons) with the alpha carbon (Cα) of the catalytic serine (S160, purple circle), defining the orientation of the active site. The vector v_1 connects the COM of *Is*PETase (yellow circle) with one of the two central carbonyl carbons ($C(=O)_A$ blue circle) of (PET)$_4$. The angle a_0 (green dashed arrow) between v_0 and v_1 was progressively reduced to 0° to align the substrate's reactive center with the enzyme's active site, while maintaining a minimum distance d_0 (green solid line) of 2.5 nm to allow the (PET)$_4$ chain to move above the protein surface. The distance d_1 (pink line) represents the separation between the central carbonyl carbon $C(=O)_A$ and the hydroxyl oxygen (Oγ) of S160, whereas d_lw (orange line) defines the distance between the terminal carbonyl carbons ($C(=O)_{ter}$). Briefly, (PET)$_4$ was first randomly inserted into the solvated box (position 1, blue dot). The angle between v_0 and v_1 was then gradually reduced to 0° to align the PET chain with the active site (position 2, blue dot), and finally the distance between $C(=O)_A$ and Oγ (S160) was decreased to 0.5 nm to generate a bound pose (position 3, blue circle). (B) From this configuration, the central terephthalic acid subunit of (PET)$_4$ containing $C(=O)_A$ was aligned with the monomeric 2-hydroxyethyl methyl terephthalate (HEMT) ligand in the 5XH3 crystal structure by minimizing the RMSD between the (PET)$_4$ segment and the reference HEMT configuration to 0 nm, as detailed in the PLUMED input provided in PLUMED-NEST (plumID:25.029). Image adapted from Falkenstein *et al*, *ACS Catal.* 2023, 13, 6919−6933.

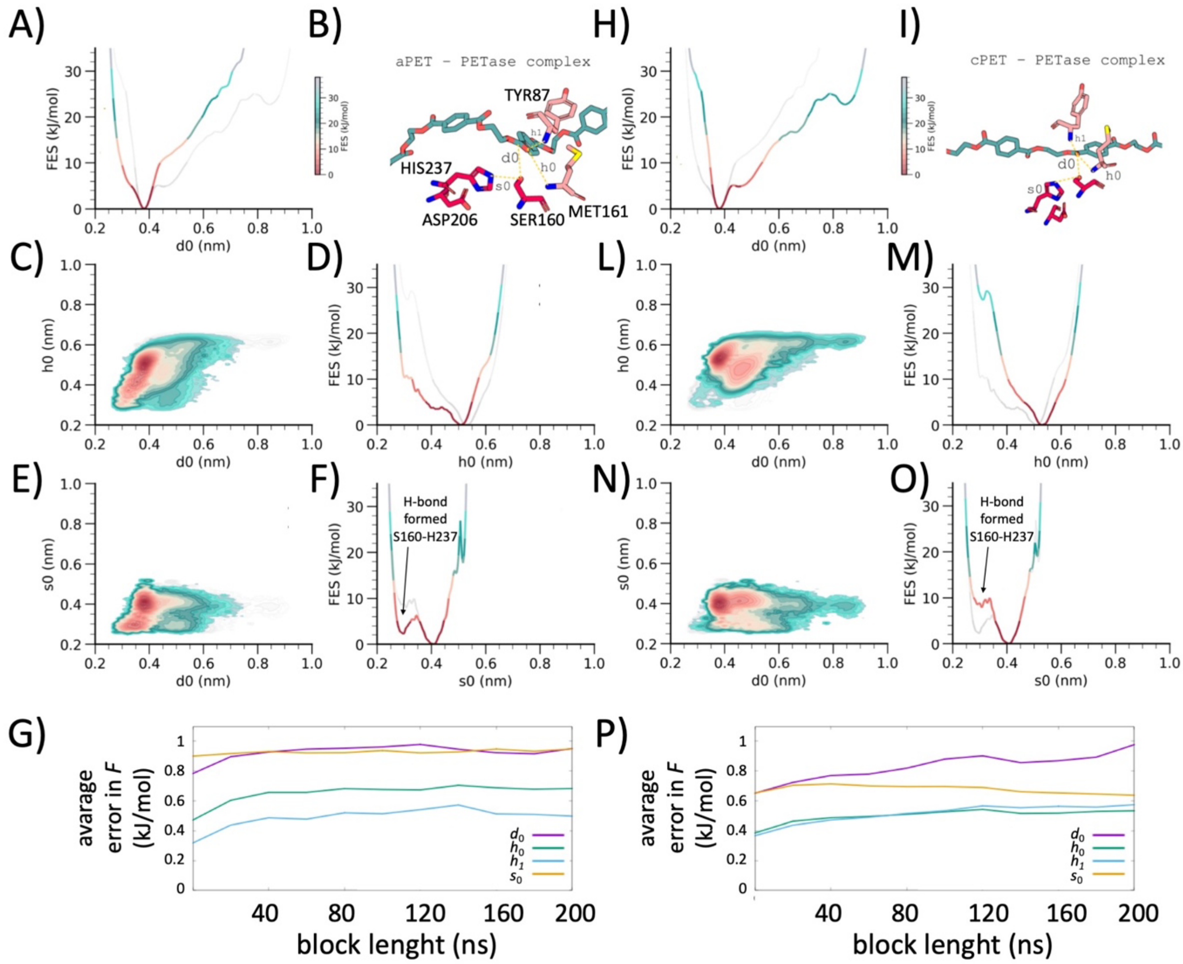

Supplementary Figure S2. Free-energy landscape of *Is*PETase–PET systems in proximity of the catalytic ensemble. Molecular model of the enzyme–PET complex showing the collective variables (CVs) used for HREX-wtMetaD analysis and reweighting with (A-G) amorphous and (H-P) crystalline PET chains. Free-energy profiles for amorphous (A) and crystalline (H) PET chains projected onto the d_0 distance (between the Oγ of catalytic Ser160 and the PET carbonyl carbon), h_0 distance (D; M), and the s_0 distance (F; O). Free-energy surfaces of *Is*PETase bound to amorphous (C) and crystalline (L) PET, shown as functions of the d_0 and h_0 distances. Free-energy surfaces of *Is*PETase bound to amorphous (E) and crystalline (N) PET, shown as functions of the d_0 and s_0 distances. In panels A, D, and F, faded gray lines indicate the equivalent profile of the crystalline PET chain; in panels H, M, and O, they indicate the corresponding profile of the modeled amorphous PET chain, shown for comparison. Estimated statistical error in the reconstructed free-energy profiles is shown as a function of block-averaging length for *Is*PETase–PET systems with (G) amorphous and (P) crystalline PET chains.

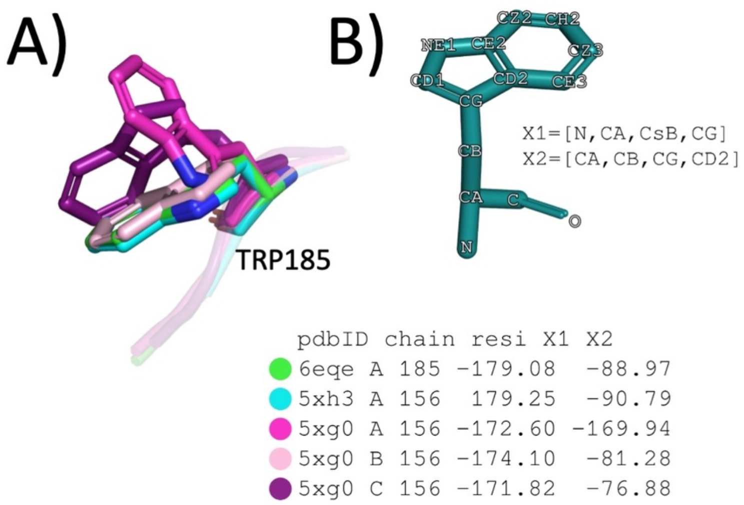

Supplementary Figure S3. Flexibility of wobbling Trp185 in *Is*PETase X-ray crystal structures. A) Comparison of χ_1 and χ_2 values across different X-ray crystal structures (PDB IDs indicated). B) Definition of the dihedral angles χ_1 and χ_2 of Trp185 used to reconstruct the free-energy landscape shown in Figure 3 of the manuscript.

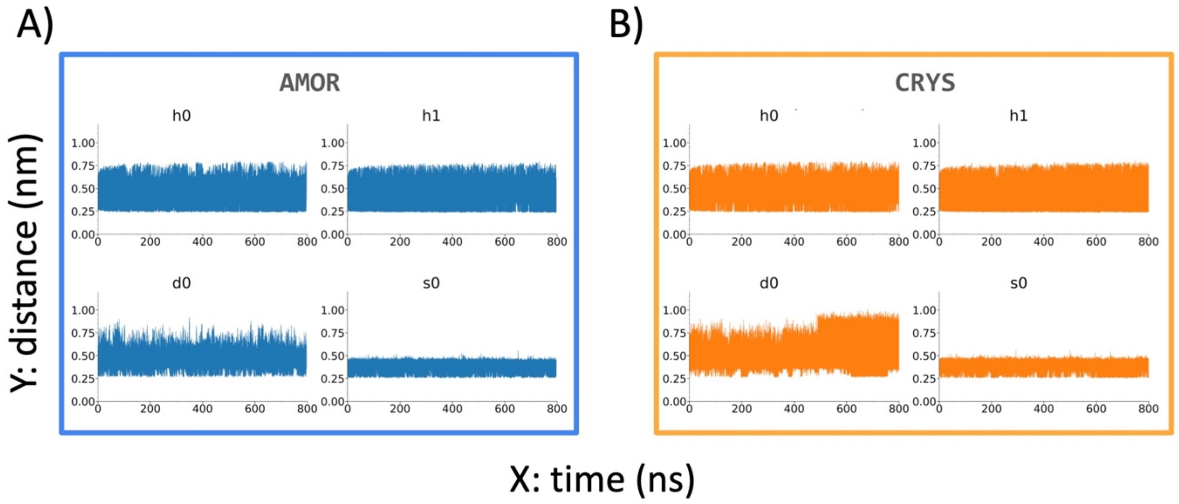

Supplementary Figure S4. Diffusion of the *Is*PETase–PET systems in the explored CV space as a function of simulation time for (A) amorphous and (B) crystalline PET chains, highlighting the enhanced exploration of the same regions multiple times in our combined HREX-wtMTD protocol.

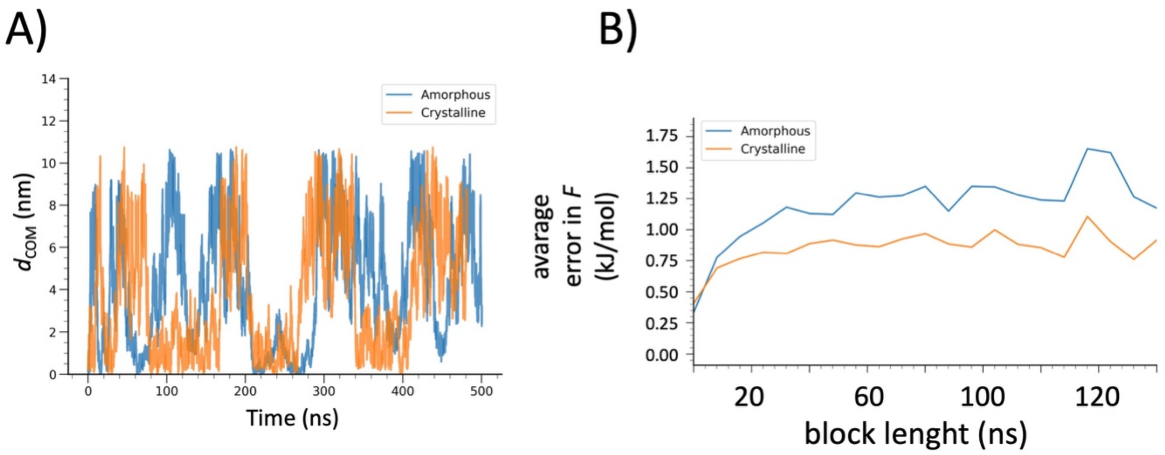

Supplementary Figure S5. (A) Evolution of the biased CV, d_{COM} , for the PET–PET systems as a function of simulation time for amorphous (blue) and crystalline (orange) PET chains, highlighting the detachment of the two chains and revisiting of the same regions multiple times during the wtMTD simulations. Note that Figure 4A of the manuscript shows multiple free-energy profiles extracted from the last 50 ns at 5 ns intervals. The traces diverge only beyond ≈ 2.5 nm for cPET and ≈ 1.2 nm for aPET. This behavior is expected: once the bound-state free-energy basins are sampled, the system increasingly explores unbound states. Consequently, repeated reconstructions produce a single dominant line in well-sampled regions, while showing greater variability in the unbound state, where biasing potentials are still applied. This trend is also evident from the time evolution of the biased CV (d_{COM}), shown above. The cPET system does not visit the bound state (d_{COM} < ≈2.5 nm) in the last 100 ns of the simulation, whereas the aPET system briefly revisits a bound state (d_{COM} ≈ 1 nm) around 450 ns. (B) Estimated statistical error in the free-energy profiles as a function of block-averaging length for amorphous (blue)and crystalline (orange) PET-PET systems.

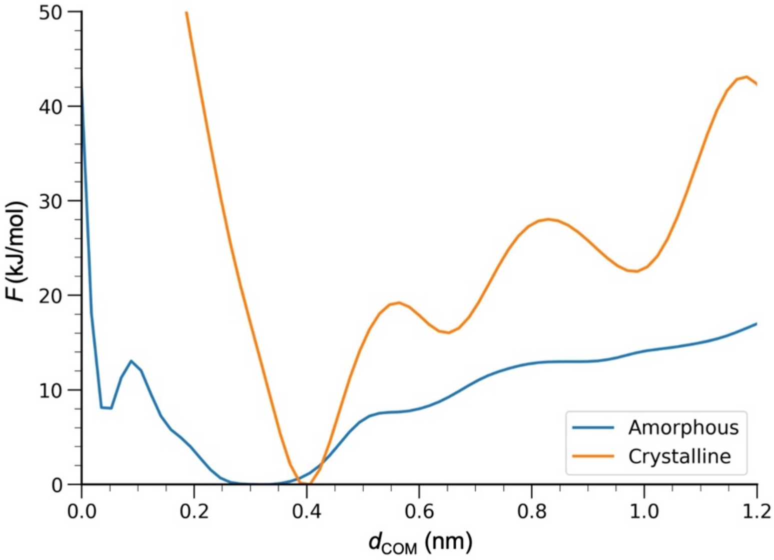

Supplementary Figure S6. Preliminary simulations of PET–PET chain detachment were carried out in a box approximately half the size of that used in Figure 4A with similar sampling time (500 ns). These simulations allowed sampling of the 0–1.2 nm region of the d_{COM} coordinate, where both aPET and cPET remain in the bound basin. The resulting preliminary profiles are fully consistent with the corresponding portion of the free-energy curves reported in Figure 4A. Free-energy profiles are shown along the distance between the centers of mass (d_{COM}) of two PET oligomers in cPET (orange) and aPET (blue).

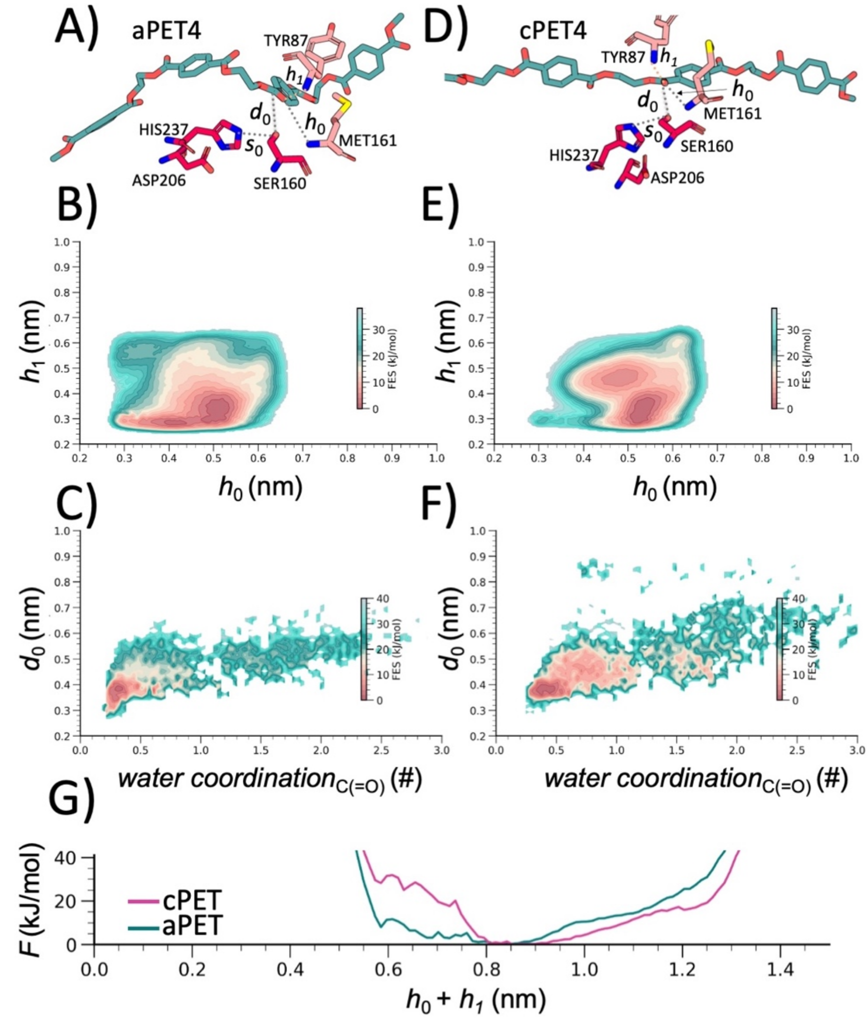

Supplementary Figure S7. Free-energy landscape of IsPETase–PET systems near the catalytic ensemble. Molecular models of the enzyme–PET complex show the collective variables (CVs) used for HREX-wtMetaD analysis and reweighting for amorphous (aPET, A–C) and crystalline (cPET, D–F) PET chains. Free-energy profiles projected onto the h_0 and h_1 distances (B, E) illustrate engagement with the oxyanion hole residues Met161 (h_0) and Tyr87 (h_1). Coordination of the PET carbonyl oxygen C(=O) with surrounding water molecules was calculated, and the free-energy surfaces were projected along this CV and the distance d_0 (between Oγ of catalytic Ser160 and the PET carbonyl carbon) for aPET (C) and cPET (F). These analyses show that, as PET approaches catalytically competent configurations at low d_0 values, surrounding water molecules are progressively depleted, consistent with the absence of water during the acylation step. (G) Free-energy profiles projected onto the sum of $h_0 + h_1$ distances for amorphous (cyan) and crystalline (violet) PET chains.
