# Supplementary material for: Why Do PETases Struggle with Crystalline PET? Catalytic Ensemble Sampling Reveals Molecular Bottlenecks
Source: J Phys Chem Lett. 2026 Apr 16;17(17):5069–78. doi: 10.1021/acs.jpclett.6c00308 (PMC13137247; doi:10.1021/acs.jpclett.6c00308)
Supplement: Supplementary file 2 [file jz6c00308_si_002.pdf]

jz-2026-00308j.R1

Name: Peer Review Information for "Why Do PETases Struggle with Crystalline PET? Catalytic Ensemble Sampling Reveals Molecular Bottlenecks"

First Round of Reviewer Comments

Reviewer: 1

Comments to the Author

The manuscript of Colizzi and co-workers describes a computational study on the limitations of the enzyme depolymerization of crystalline PET (cPET) based on the comparison of known experimental conformational ratios of crystalline and amorphous PET chains with enhanced-sampling molecular dynamics simulations. According to the results on Ideonella sakaiensis PETase, authors claim that the formation of catalytic ensembles of non covalent enzyme:substrate catalytic configurations of cPET is strongly hindered by the enzyme shape and dynamics, with additional energetic costs required to separate crystalline chains to fit the active site. The conclusions provide guides for engineering more efficient PETases for depolymerization of cPET

In my opinion, despite the calculations look properly carried out, and the subject of the study is of interest, my major concern is whether the results (the differences between cPET and aPET) are robust enough to support the conclusions. In particular, I have the following comments/concerns I would like authors to discuss/address:

1) As mentioned by the authors, the limitations of depolymerizing cPET is a combination of the free-energy cost of forming productive catalytic configurations for crystalline compared to amorphous PET, which depends on the structure and dynamics of the hydrolase active site, and the cost of separating tightly packed cPET chains. In this regard, I wonder if both problems are quantitatively estimated at the same level of accuracy.

2) I also have some concerns about the differences in the FEL for the cPET and aPET. It looks like the differences are in the order of some kJ/mol, which I wonder if they can explain

the experimental evidence that Is PETase is inactive against cPET. I agree that there is a trend, but I wonder if the difference is too small. I am afraid I was not able to follow the arguments and numbers in the text, when comparing with the analysis of Figure 2, especially Figure 2E.

3) Related with previous comment, I am a bit puzzle with the projection of the FESs of Fig 2C and 2D, on Figure 2E. Are they projected with a fixed h0 distance? And how were the profiles on panel F obtained? In this regard, I am also a bit skeptic on the energetic differences (1.4 kcal/mol) to justify the experimentally observed differences

4) I wonder if the argument based on pi-pi interactions employed to study the binding is robust enough, considering the limitation of the classical FF in describing this kind of interactions. Anyway, they obtain a more dramatic difference between the two cPET and aPET in this analysis.

5) Related with previous comment, I miss a deeper discussion on the values of the conformational angle of TRP185 of the different enzyme structures (PDB codes). Despite the values of the different crystal structures, how flexible are they?

6) I have a couple of questions related to the PET chain detachment. First, have authors tried to repeat the calculations with different parameters (Gaussian heights, width, length of MD, ...). I am not sure that panel C of Figure 4 shows a convergence after 400 ns, despite it is much better than at shorter times. I find a bit questionable that only from 2.5 nm (cPET) or from 1.2 nm (aPET) authors obtain more than one line. By all means, I would never report energy values with decimal places. And second; are the cartoons on panel 4B well done? (i.e. are the two oligomers chains separated by ca. 2.8 nm in the last, h, plot?).

7) Finally, I find very interesting, and honest, the limitations of the simulations discussed by the authors in the conclusions section.

Reviewer: 2

Comments to the Author

The authors study the interaction between PETase, a promising enzyme for PET degradation, and a PET tetramer prototype substrate in both its crystalline and amorphous forms. The authors applied geometrical constraints to reproduce trans/gauche ratios observed experimentally for the crystalline and amorphous forms, and combined

Hamiltonian-exchange and well-tempered metadynamics to study the interaction between PETase and the modelled PET. Furthermore, the authors also study the free energetics of PET unbinding in amorphous and crystalline forms using well-tempered metadynamics.

The authors provide evidence that formation of catalytic conformations between PETase and amorphous PET is thermodynamically more favored than for crystalline PET. They also observe that the interaction between amorphous PET fragments is less favored than for crystalline ones. The work is technically sound and may constitute an approach to inspect enzyme:polymer interactions for enzyme engineering.

However, it is my opinion that several aspects should be addressed before the work can be considered for publication at the Journal of Physical Chemistry Letters.

Major comments:

The abstract refers that the model under study “highlights limitations of current α/β-hydrolase scaffolds used for PET depolymerization”. However, the results discussed concern the interaction between a PET tetramer and the active site of PETase, or the unbinding of PET tetramers in amorphous and crystalline states. A brief mention to differences between IsPETase and HiC (another α/β-hydrolase) is discussed at the binding site level in the conclusions section, so it is my interpretation that there is no significant discussion on the repercussions of the α/β-hydrolase fold in PET degradation. The authors should provide further clarification on this point.

It is not clear how the protocol can be easily generalized to the other semicrystalline polymers, because only one case study is described. Hence, I believe the statement “Overall, the computational strategy introduced here offers a generalizable tool for probing enzyme architectures capable of accessing and activating the reactive centers of semicrystalline polymers.” in the conclusions section should be revisited.

In page 7, lines 33-35, the authors quote that “Indeed, the capacity of a chain segment at the surface to temporarily leave the solid structure and fit into the hydrolase active site is a key determinant of catalysis”. Having that in mind, I think that the choice of the PET tetramer as a substrate should be further justified, as it is quite a minimal representation of crystalline PET. The authors raise awareness of this limitation in the conclusions section, but it is my opinion that the choice of PET chain length should be more properly supported, as it is not sufficiently convincing why the results on the unbinding of such small monomers can be transferred to an actual PET surface/melt.

Small comments:

In Figure 1, I would suggest that the conformational distribution of the EG torsion angles to be represented for more spaced distances d1, because panel E is difficult to interpret.

In Figure 2, panels C-D, mechanistic studies show that efficient catalysis by PETase requires both distances h0 and h1, so I would suggest that the authors tried to explore the free energy surface representation as a function of the sum h0+h1. Availability of water in the active site should also be considered when considering the categorization of reactive conformations as no water molecules are typically at the active site of PETase during the acylation step. In panels E-F, colors in the legend don't seem to match those of the free energy plots (in particular for cPET).

In page 6, line 17, the equation "population ratio=e^(ΔF/kBT))" should be written as an equation object for clarity.

In page 6, line 27, my interpretation is that "The stability of the H-bond between Ser160 and His237, identified by a minimum in the free-energy profile at $s_0 \approx 0.3$ nm, is about 6 kJ/mol higher when the enzyme interacts with cPET than with aPET chains (Figure 2F)."; please clarify.

Figure 4, panel C, does the time axis correspond to accumulated time considered for the free energy calculation? Please clarify.

In the methods section, page 12, lines 12-13, why did the authors opted for such a large solvated water box (12 nm) to simulate the detachment of the PET monomers?

Author's Response to Peer Review Comments:

Barcelona, March 20, 2026

Dear Editor:

Please find enclosed the revised version of manuscript entitled "Why Do PETases Struggle with Crystalline PET? Catalytic Ensemble Sampling Reveals Molecular Bottlenecks" that we have carefully revised according to the suggestion of the reviewers. The reviewers' and editorial

comments were fully implemented in the manuscript, which we think meets now the high-level criteria required for publication in *The Journal of Physical Chemistry Letters.*

A point-by-point response to the issues raised by the reviewers is provided below.

With kind regards,

Francesco Colizzi

Institute for Advanced Chemistry of Catalonia, IQAC-CSIC

Spanish National Research Council, CSIC

Email: colizzi@csic.es

We thank the Reviewers for carefully reading the manuscript and for their comments and suggestions, which provided an opportunity to revise the manuscript and improve its overall quality. We have thoroughly edited the manuscript accordingly. Below is a point-by-point reply to the

Reviewers' suggestions, with our responses in bold text

Reviewer: 1

Recommendation: This paper may be publishable, but major revision is needed; I would like to be invited to review any future revision.

Comments:

The manuscript of Colizzi and co-workers describes a computational study on the limitations of the enzyme depolymerization of crystalline PET (cPET) based on the comparison of known experimental conformational ratios of crystalline and amorphous PET chains with enhanced-sampling molecular dynamics simulations. According to the results on Ideonella sakaiensis PETase, authors claim that the formation of catalytic ensembles of non covalent enzyme:substrate catalytic configurations of cPET is strongly hindered by the enzyme shape and dynamics, with additional energetic costs required to separate crystalline chains to fit the active site. The conclusions provide guides for engineering more efficient PETases for depolymerization of cPET.

In my opinion, despite the calculations look properly carried out, and the subject of the study is of interest, my major concern is whether the results (the differences between cPET and aPET) are robust enough to support the conclusions. In particular, I have the following comments/concerns I would like authors to discuss/address:

1) As mentioned by the authors, the limitations of depolymerizing cPET is a combination of the freeenergy cost of forming productive catalytic configurations for crystalline compared to amorphous PET, which depends on the structure and dynamics of the hydrolase active site, and the cost of separating tightly packed cPET chains. In this regard, I wonder if both problems are quantitatively estimated at the same level of accuracy.

We thank the reviewer for highlighting this point. Both the catalytic ensemble and PET chain separation free energies were computed using metadynamics-based techniques with the same force field, water model, and PET chain length (tetramer). This setup was intended to preserve consistency between the two estimates and to ensure that numerical errors, as

well as forcefield limitations, affect both quantities in a comparable manner. We acknowledge that the prevalent stacking interactions in the PET–PET dimerization simulations, together with the approximations of the force field, may influence the absolute accuracy of these estimations. Despite these structural differences, this setup still allows a reasonable comparison, as also supported by the block-averaged free-energy profiles (Supporting Figures S2 and S5), which report the average error and show consistent behavior across the simulations.

In response to the Reviewer's comments, we have expanded the discussion of these aspects and have extended the limitations section in the Conclusions (page 10).

2) I also have some concerns about the differences in the FEL for the cPET and aPET. It looks like the differences are in the order of some kJ/mol, which I wonder if they can explain the experimental evidence that Is PETase is inactive against cPET. I agree that there is a trend, but I wonder if the difference is too small. I am afraid I was not able to follow the arguments and numbers in the text, when comparing with the analysis of Figure 2, especially Figure 2E.

We thank the reviewer for this comment and for pointing out the need to clarify the interpretation of Figure 2, especially panel E, in the manuscript. We acknowledge that some free-energy differences visible on the 2D surfaces (Figures 2C–D) are only a few kJ/mol. To improve clarity, we have extensively revised this section (starting on page 4 of the revised manuscript), redesigned Figure 2 (expanding panel E and moving panel D to the Supporting Information, now included only as Supplementary Figures 2F and 2O), and more clearly distinguished between (i) small local variations between neighboring basins and (ii) the larger free-energy penalty that crystalline versus amorphous PET must overcome to reach catalytically competent configurations.

The relevant comparison is the *free-energy difference leading to productive geometries* along the d_0 coordinate (Figure 2E). In Figure 2E, at the catalytic distance $d_0 \approx 0.30$ nm required for nucleophilic attack, the minimum free-energy for crystalline PET is about 25 kJ/mol higher than for amorphous PET. This is the main energy difference discussed in this section, which we now emphasize to distinguish it from the smaller local basin variations. Also, as now further clarified in the revised manuscript (page 6), "This [≈25 kJ/mol] energetic penalty in the reactant state of cPET relative to aPET reduces the population of catalytically competent complexes by ~24000-fold (calculated as $\boldsymbol{e}^{!\Delta F/k_\mathrm{B}T}$, where $\Delta\boldsymbol{F}$ is the free-energy difference, $\boldsymbol{k}_\mathbf{B}$ the Boltzmann constant, and $\boldsymbol{T}$ the temperature). This drastic depletion effectively eliminates productive cPET-bound catalytic ensembles, in line with the near-

complete loss of enzymatic activity. By comparison, literature shows that even a few orders-of-magnitude decreases in the population of catalytically competent substates can lead to substantial activity drops."

3) Related with previous comment, I am a bit puzzle with the projection of the FESs of Fig 2C and 2D, on Figure 2E. Are they projected with a fixed h0 distance? And how were the profiles on panel F obtained? In this regard, I am also a bit skeptic on the energetic differences (1.4 kcal/mol) to justify the experimentally observed differences.

We thank the reviewer for highlighting a point that required further clarification. The free-energy profiles shown in Figure 2E are not computed at a fixed h_0 value. Instead, the 1D profiles represent the unbiased marginal free energies, obtained by reweighting the metadynamics-biased trajectories and integrating over all other degrees of freedom, including h_0. Accordingly, Figure 2E shows the 1D free energy profile by projecting the full free energy surface onto d_0 rather than a slice at fixed h_0.

Regarding panel F—now moved to the Supplementary Material to emphasize the main message of Figure 2—the profiles were obtained using the same procedure and represent the marginal free energy, obtained by integrating over all remaining coordinates. The energetic difference of ~6 kJ/mol observed in panel F does not correspond to the penalty for forming catalytically competent enzyme–substrate complexes. That dominant contribution is the ~25 kJ/mol difference shown in Figure 2E. Panel F instead reflected the relative stability of the Ser160–His237 hydrogen bond once the substrate is already positioned in the active site—a secondary effect compared to the main catalytic competence penalty.

Because panel F is mechanistically supportive but not central to the main message of Figure 2, we have revised the Figure and moved panel F to the Supporting Information (Supplementary Figures S2F and S2O) for clarity.

4) I wonder if the argument based on pi-pi interactions employed to study the binding is robust enough, considering the limitation of the classical FF in describing this kind of interactions. Anyway, they obtain a more dramatic difference between the two cPET and aPET in this analysis.

We thank the reviewer for this comment. We agree that classical force fields have limitations in describing absolute π–π interaction energies. However, the analysis in Figure 3 does not aim to provide a quantitative estimate of these interactions. Instead, it

illustrates how the rigidity of cPET modulates the conformational space of Trp185, a residue known from multiple PETase crystal structures to adopt distinct “wobbling” orientations during substrate accommodation. Thus, Figure 3 highlights Trp185 conformational gating as the structural origin of the differences between aPET and cPET, in agreement with experimental observations, rather than making claims about the detailed energetics of π–π interactions.

Regarding PET–PET detachment, as noted by the reviewer, although the force field provides only an approximate description of π–π interactions, the same approximation is consistently applied to both systems. This allows the simulations to capture relative differences in population that are directly related to the distinct conformational behavior and packing geometries of cPET and aPET.

Following the reviewer comment, we have extensively revised the text (pages 6 and 10) and edited the Figure 3 legend to clarify this point.

5) Related with previous comment, I miss a deeper discussion on the values of the conformational angle of TRP185 of the different enzyme structures (PDB codes). Despite the values of the different crystal structures, how flexible are they?

The χ_2 angles indicated in Figure 3 correspond to values observed across several *Is*PETase crystal structures. In these X-ray structures, the side chain of Trp185 spans a broad range of χ_2 values, indicating high flexibility. Mutation studies (Refs. 41 and 42 in the revised manuscript) further suggest that this flexibility is functionally important for binding and depolymerizing aPET.

Our simulations reproduce this experimental behavior. In the aPET complex, Trp185 samples the same χ_2 region observed in the crystal structures, and the MD ensemble extends this space smoothly while remaining within the same rotameric basins. In the cPET complex, in addition to the experimentally observed χ_2 values, Trp185 populates an additional flipped rotamer ($\chi_2 \approx +90°$, Figure 3B) that, to the best of our knowledge, has not been reported in available PETase structures. This flipped state connects to configurations compatible with productive binding, but only through high free-energy regions of the landscape.

We thank the reviewer for highlighting the importance of this point, which we have now incorporated in the discussion of Figure 3 in the revised manuscript (page 6) and further detailed in Supplementary Figure S3.

6) I have a couple of questions related to the PET chain detachment. First, have authors tried to repeat the calculations with different parameters (Gaussian heights, width, length of MD, …). I am not sure that panel C of Figure 4 shows a convergence after 400 ns, despite it is much better than at shorter times. I find a bit questionable that only from 2.5 nm (cPET) or from 1.2 nm (aPET) authors obtain more than one line. By all means, I would never report energy values with decimal places. And second; are the cartoons on panel 4B well done? (i.e. are the two oligomers chains separated by ca. 2.8 nm in the last, h, plot?).

We thank the reviewer for these constructive observations.

(i) As standard practice, we performed several preliminary well-tempered metadynamics simulations using different combinations of Gaussian height, width, deposition pace, and bias factor before selecting the parameters used in the production runs. Because the biased collective variable is a simple distance (d_{COM}, the COM–COM separation between the two PET oligomers), the results were robust to these parameter variations. In addition, we repeated the simulations multiple times while progressively enlarging the simulation box to ensure that full chain detachment could be sampled without artificial boundary effects.
As shown in the new Supporting Figure S6, simulations carried out in a box approximately half the size of the one used in Figure 4 allowed sampling of the 0–1.2 nm region of the d_{COM} coordinate (where both aPET and cPET remain in the bound basin), and these preliminary profiles are fully consistent with the corresponding portion of the free-energy curves reported in Figure 4A. However, only simulations performed in a sufficiently large box permitted complete sampling of the fully detached states.

(ii) We appreciate the reviewer request for clarification about the convergence of the estimations. In the manuscript, we observed that the relative ΔΔF between aPET and cPET stabilizes after about 400 ns, converging to an approximate 35 kJ/mol difference. We have now revised Figure 4C to explicitly show the cumulative estimate of ΔΔF (black line), which converge to ~35 kJ/mol within the last part of the simulation. The individual ΔF estimations for aPET and cPET are inherently more difficult to converge because sampling the unbound region can continue to reveal new microstates over long timescales. The slow drift in the bound–unbound free-energy difference therefore reflects incomplete sampling of the unbound basin, and we have clarified this point in the revised text (page 8).

(iii) Figure 4A shows multiple free-energy profiles extracted from the last 50 ns at 5 ns intervals. The reviewer correctly notes that these traces diverge only beyond ≈ 2.5 nm for cPET and ≈ 1.2 nm for aPET. This behavior is expected: after sampling several binding and unbinding events and filling the free-energy basins related to bound states, the system increasingly spends more time visiting unbound states (as mentioned in point ii). Repeated reconstructions therefore, naturally produce a single dominant line in the region where the sampling has been more thorough, while showing greater variability in the unbound state, where biasing potentials are still applied.

This behavior is also evident from the time evolution of the biased CV (d_{COM}), shown in Supplementary Figure S5A, which indicates that the cPET system does not visit the bound state (d_{COM} < ≈2.5 nm) in the last 100 ns of the simulations, while the aPET system briefly revisits a bound state (d_{COM} ≈ 1 nm) around 450 ns. We have clarified this point in the caption of Supplementary Figure S5A, which is now also referenced in the legend of Figure 4A.

(iv) We agree that reporting decimal places may imply unwarranted precision. In the revised manuscript, all free-energy values are now rounded to integer kJ/mol.

(v) Accuracy of the cartoons in Figure 4B. The ≈2.8 nm value refers specifically to the center-of-mass distance, d_{COM}, between the two oligomers, not to the minimal atom–atom separation. We have checked the structural frames used to generate the cartoons and confirm that they correspond to typical configurations at the indicated d_{COM} values prior to complete chain separation. We have clarified this point in the legend of Figure 4B.

7) Finally, I find very interesting, and honest, the limitations of the simulations discussed by the authors in the conclusions section.

We thank the Reviewer for this positive feedback. We appreciate the acknowledgement of our effort to explicitly discuss the limitations of our work.

Reviewer: 2

Recommendation: This paper may be publishable, but major revision is needed; I would like to be invited to review any future revision.

Comments:

The authors study the interaction between PETase, a promising enzyme for PET degradation, and a PET tetramer prototype substrate in both its crystalline and amorphous forms. The authors applied geometrical constraints to reproduce trans/gauche ratios observed experimentally for the crystalline and amorphous forms, and combined Hamiltonian-exchange and well-tempered metadynamics to study the interaction between PETase and the modelled PET. Furthermore, the authors also study the free energetics of PET unbinding in amorphous and crystalline forms using well-tempered metadynamics.

The authors provide evidence that formation of catalytic conformations between PETase and amorphous PET is thermodynamically more favored than for crystalline PET. They also observe that the interaction between amorphous PET fragments is less favored than for crystalline ones. The work is technically sound and may constitute an approach to inspect enzyme:polymer interactions for enzyme engineering.

However, it is my opinion that several aspects should be addressed before the work can be considered for publication at the Journal of Physical Chemistry Letters.

Major comments:
-The abstract refers that the model under study "highlights limitations of current α/β-hydrolase scaffolds used for PET depolymerization". However, the results discussed concern the interaction between a PET tetramer and the active site of PETase, or the unbinding of PET tetramers in amorphous and crystalline states. A brief mention to differences between IsPETase and HiC (another α/βhydrolase) is discussed at the binding site level in the conclusions section, so it is my interpretation that there is no significant discussion on the repercussions of the α/β-hydrolase fold in PET degradation. The authors should provide further clarification on this point.

We thank the reviewer for pointing out that this aspect needed clarification. Our intention was to highlight that specific active-site features commonly found in α/βhydrolases impose intrinsic geometrical constraints on how amorphous and crystalline PET chains can approach the catalytic center and form catalytically competent configurations.

We agree that, in the original abstract, this point was presented too generally, and that the manuscript did not clearly distinguish between limitations arising from the active-site architecture typical of α/β-hydrolase PETases and broader implications for the α/β-hydrolase fold as a whole.

We have therefore reformulated the sentence in the abstract to more precisely reflect what our data support. The revised abstract now reads: “The model highlights limitations of the active-site architectures typically found in α/β-hydrolase scaffolds used for PET depolymerization.”

-It is not clear how the protocol can be easily generalized to the other semicrystalline polymers, because only one case study is described. Hence, I believe the statement “Overall, the computational strategy introduced here offers a generalizable tool for probing enzyme architectures capable of accessing and activating the reactive centers of semicrystalline polymers.” in the conclusions section should be revisited.

We thank the reviewer for this observation. We agree that, as presented, our original conclusion overstated the generalizability of the protocol, given that the manuscript focuses on a single case study involving PET. Following reviewer suggestion, we have revised the concluding statement to avoid implying an untested level of generality. The sentence (page 10) now reads: “Overall, the computational strategy introduced here provides a framework for probing how enzyme active-site architectures interact with substrates that display both crystalline and amorphous conformational states.”

-In page 7, lines 33-35, the authors quote that “Indeed, the capacity of a chain segment at the surface to temporarily leave the solid structure and fit into the hydrolase active site is a key determinant of catalysis”. Having that in mind, I think that the choice of the PET tetramer as a substrate should be further justified, as it is quite a minimal representation of crystalline PET. The authors raise awareness of this limitation in the conclusions section, but it is my opinion that the choice of PET chain length should be more properly supported, as it is not sufficiently convincing why the results on the unbinding of such small monomers can be transferred to an actual PET surface/melt.

We thank the reviewer for raising this important point. We agree that the binding and unbinding of two PET oligomers—regardless of their length—represents a minimal structural unit compared to a complete PET surface/melt. The rationale for using a short oligomer (a tetramer in our case) is that it still preserves the key local structural features of crystalline and amorphous PET as modeled here, namely the experimentally determined trans/gauche conformational ratios and the resulting interchain packing. In this context, the tetramer—which was also used in our catalytic PET–PETase ensemble estimations—

represents a simplified system that provides a conceptual framework to compare the energetics of catalytic ensemble formation with an estimate of the energetic cost required to locally extract a PET segment from amorphous (aPET) versus crystalline (cPET) environments.

It is interesting to note that the resulting approximate detachment costs of ~19 kJ/mol per monomer for cPET and ~11 kJ/mol per monomer for aPET fall within the range of experimental estimates derived from lamellar fold surface free energies (~25 kJ/mol per repeat unit).

We fully agree, however, that free-energy estimations derived from tetramers (un)binding cannot be interpreted as descriptors of detachment from an extended PET surface. In the revised text (page 10), we now clarify that these unbinding results should be viewed as indicators of the relative ease with which aPET versus cPET chain ends can disengage from their local environments, rather than as a full representation of polymer-surface energetics. We further comment on these limitations in the Conclusions section (page 10).

Small comments:
-In Figure 1, I would suggest that the conformational distribution of the EG torsion angles to be represented for more spaced distances d1, because panel E is difficult to interpret.

In the revised version of the manuscript, we have updated Figure 1E. The EG torsion angle distributions are now shown at more widely spaced d_1 intervals, and fewer than half of the original lines are displayed, which greatly improves visual clarity and readability. We thank the reviewer for this suggestion

-In Figure 2, panels C-D, mechanistic studies show that efficient catalysis by PETase requires both distances h0 and h1, so I would suggest that the authors tried to explore the free energy surface representation as a function of the sum h0+h1. Availability of water in the active site should also be considered when considering the categorization of reactive conformations as no water molecules are typically at the active site of PETase during the acylation step. In panels E-F, colors in the legend don't seem to match those of the free energy plots (in particular for cPET).

We thank the reviewer for this suggestion. In Supplementary Figure S7, we now include free-energy surfaces as a function of both h_0 and h_1, showing the engagement of aPET and

cPET with the oxyanion hole residues Tyr87 (h_1) and Met161 (h_0). Also, the free-energy profile as a funcion of the sum $h_0 + h_1$ is shown (Supplementary Figure S7G).

We also calculated the coordination of the carbonyl oxygen C(=O) of PET with water molecules and projected the free-energy surface along this collective variable and the distance d_0 from the catalytic Ser160 (Supplementary Figure S7C-F). This analysis shows that, as PET approaches the catalytically competent configurations, there is a marked reduction in the number of surrounding water molecules, consistent with the absence of water during the acylation step. Water coordination indicates that, as a catalytically competent configuration is formed, water is progressively depleted. Label colors in Figure 2 have also been corrected.

-In page 6, line 17, the equation “population ratio=e^(ΔF/kBT))” should be written as an equation object for clarity.

The population ratio is now written as an equation object.

-In page 6, line 27, my interpretation is that “The stability of the H-bond between Ser160 and His237, identified by a minimum in the free-energy profile at $s_0 \approx 0.3$ nm, is about 6 kJ/mol higher when the enzyme interacts with cPET than with aPET chains (Figure 2F).”; please clarify.

We thank the reviewer for pointing out that this sentence required clarification. We have revised the entire paragraph in the manuscript (page 6), which now reads: “Furthermore, we observed that the substrate conformational state modulates the stability of the hydrogen bond between Ser160 and the adjacent His237, which is essential for Ser160 activation for nucleophilic attack (distance s_0; Figure 2A–B and Supplementary Figures 2F and 2O). In particular, in the presence of cPET, the Ser160–His237 hydrogen bond is approximately 6 kJ/mol less stable than in the aPET complex

(Supplementary Figures 2F and 2O).”

Because panel 2F provides mechanistic support but is not central to the main message of Figure 2, we have revised the corresponding text, updated the figure, and moved panel F to the Supplementary Information (Supplementary Figures 2F and 2O) to improve clarity.

-Figure 4, panel C, does the time axis correspond to accumulated time considered for the free energy calculation? Please clarify.

Yes. The time axis in Figure 4C represents the accumulated simulation time used for the calculation of free-energy differences; the legend has been updated accordingly.

-In the methods section, page 12, lines 12-13, why did the authors opted for such a large solvated water box (12 nm) to simulate the detachment of the PET monomers?

The large solvated water box was chosen to allow full sliding and detachment of the PET oligomers, particularly cPET, while avoiding boundary artifacts. We have clarified this point in the corresponding Methods section (page 12).

jz-2026-00308j.R2

Name: Peer Review Information for "Why Do PETases Struggle with Crystalline PET? Catalytic Ensemble Sampling Reveals Molecular Bottlenecks"

Second Round of Reviewer Comments

Reviewer: 1

Comments to the Author

Authors have improved the manuscript, addressing most of my original concerns and answering my questions. Consequently, I recommend publishing the manuscript as it is.

Reviewer: 2

Comments to the Author

In the revised version of the manuscript, the authors performed additional analysis to address the points raised by the reviewers. Overall, the authors properly addressed the comments given, and the revised version has improved significantly.

Personally, it is my opinion that the work would still improve significance if other enzymes with the same fold were included in the study to make the protocol more relevant and transferable. Nevertheless, the manuscript still reports novel research on enzymatic PET degradation, the research topic is timely and the methodology is technically very sound.

Hence, in my opinion, the final version of the manuscript is suitable for publication in the Journal of Physical Chemistry Letters, after the minor revisions noted:

Minor revisions:

Figure S2, I suggest that the caption is extended to make explicit what the faded lines in panels A, D, F, H, M, O correspond to. In addition, I could not figure the meaning of the arrow pointing downwards in panels A, D, H and M.

Figure S7, panel G, I believe the colors of the legend don't match the color of the curves; please verify.

Author's Response to Peer Review Comments:

Dear Editor:

Please find enclosed the revised version of the manuscript entitled "Why Do PETases Struggle with Crystalline PET? Catalytic Ensemble Sampling Reveals Molecular Bottlenecks", which has been further revised in response to the minor comments of the reviewers. All reviewers and editorial suggestions have been addressed and implemented in the manuscript. We hope that the revised version now meets the high standards required for publication in The Journal of Physical Chemistry Letters.

A point-by-point response to the issues raised by the reviewers is provided in the Cover Letter and also attached in a separate file.

With kind regards,

Francesco Colizzi

Institute for Advanced Chemistry of Catalonia, IQAC-CSIC

Spanish National Research Council, CSIC

Email: colizzi@csic.es

Reviewer: 1

Recommendation: This paper represents a significant new contribution and should be published as is.

Comments: Authors have improved the manuscript, addressing most of my original concerns and answering my questions. Consequently, I recommend publishing the manuscript as it is.

We thank the reviewer for acknowledging the improvements made in the revised manuscript and for recommending its publication in its current form.

Reviewer: 2

Recommendation: This paper is publishable subject to minor revisions noted. Further review is not needed.

Comments: In the revised version of the manuscript, the authors performed additional analysis to address the points raised by the reviewers. Overall, the authors properly addressed the comments given, and the revised version has improved significantly.
Personally, it is my opinion that the work would still improve significance if other enzymes with the same fold were included in the study to make the protocol more relevant and transferable. Nevertheless, the manuscript still reports novel research on enzymatic PET degradation, the research topic is timely and the methodology is technically very sound.

Hence, in my opinion, the final version of the manuscript is suitable for publication in the Journal of Physical Chemistry Letters, after the minor revisions noted:

We thank the reviewer for the careful evaluation of the revised manuscript and for the positive assessment of our responses to the reviewers. We appreciate the suggestion to extend the protocol to additional enzymes with the same fold, which we agree would be a valuable direction for future work aimed at broadening transferability. We are grateful to the reviewer for recognizing the novelty, timeliness, and technical soundness of the present study, and for recommending the manuscript for publication in *The Journal of Physical Chemistry Letters*.

Minor revisions:

-Figure S2, I suggest that the caption is extended to make explicit what the faded lines in panels A, D, F, H, M, O correspond to. In addition, I could not figure the meaning of the arrow pointing downwards in panels A, D, H and M.

The faded lines in Figure S2 have now been explicitly defined, and the following sentence has been added to the legend: "In panels A, D, and F, faded gray lines indicate the equivalent profile of the crystalline PET chain; in panels H, M, and O, they indicate the corresponding profile of the modeled amorphous PET chain, shown for comparison."
The downward arrows were indeed misleading and have now been removed. We thank the reviewer for bringing this to our attention.

-Figure S7, panel G, I believe the colors of the legend don't match the color of the curves; please verify.

We thank the reviewer for highlighting this color mismatch, which has now been corrected.
